# Four irradiation and three positioning techniques for whole‐breast radiotherapy: Is sophisticated always better?

**DOI:** 10.1002/acm2.13720

**Published:** 2022-09-15

**Authors:** Max Schoepen, Bruno Speleers, Wilfried De Neve, Vincent Vakaet, Pieter Deseyne, Leen Paelinck, Annick Van Greveling, Liv Veldeman, Jan Detand, Werner De Gersem

**Affiliations:** ^1^ Department of Human Structure and Repair, Faculty of Medicine and Health Sciences Ghent University Ghent Belgium; ^2^ Department of Industrial Systems Engineering and Product Design, Faculty of Engineering and Architecture Ghent University Kortrijk Belgium; ^3^ Department of Radiation Oncology University Hospital Ghent Ghent Belgium

**Keywords:** breast cancer, DIBH, IMAT, multi‐beam, prone, prone crawl, radiotherapy, supine, tangential fields

## Abstract

**Purpose:**

We report on a dosimetrical study of three patient positions (supine, prone dive, and prone crawl) and four irradiation techniques for whole‐breast irradiation (WBI): wedged‐tangential fields (W‐TF), tangential‐field intensity‐modulated radiotherapy (TF‐IMRT), multi‐beam IMRT (MB‐IMRT), and intensity‐modulated arc therapy (IMAT). This is the first study to evaluate prone crawl positioning in WBI and the first study to quantify dosimetrical and anatomical differences with prone dive positioning.

**Methods:**

We analyzed five datasets with left‐ and right‐sided patients (*n* = 51). One dataset also included deep‐inspiration breath hold (DIBH) data. A total of 252 new treatment plans were composed. Dose–volume parameters and indices of conformity were calculated for the planning target volume (PTV) and organs‐at‐risk (OARs). Furthermore, anatomical differences among patient positions were quantified to explain dosimetrical differences.

**Results:**

Target coverage was inferior for W‐TF and supine position. W‐TF proved overall inferior, and IMAT proved foremost effective in supine position. TF‐IMRT proved competitive to the more demanding MB‐IMRT and IMAT in prone dive, but not in prone crawl position. The lung‐sparing effect was overall confirmed for both prone dive and prone crawl positioning and was largest for prone crawl. For the heart, no differences were found between prone dive and supine positioning, whereas prone crawl showed cardiac advantages, although minor compared to the established heart‐sparing effect of DIBH. Dose differences for contralateral breast were minor among the patient positions. In prone crawl position, the ipsilateral breast sags deeper and the PTV is further away from the OARs than in prone dive position.

**Conclusions:**

The prone dive and prone crawl position are valid alternatives to the supine position in WBI, with largest advantages for lung structures. For the heart, differences are small, which establishes the role of DIBH in different patient positions. These results may be of particular interest to radiotherapy centers with limited technical resources.

## 1 INTRODUCTION

### 1.1 Background

Radiotherapy (RT) after breast‐conserving surgery improves locoregional control and survival. These benefits may occur at the expense of dose‐dependent acute and late toxicity, such as pneumonitis, fibrosis, ischemic heart disease, skin changes, and radiation‐induced cancers.^1^, ^2^, ^3^, ^4^ Strategies for sparing organs‐at‐risk (OARs), while ensuring adequate target coverage, are therefore essential. Different approaches can be implemented and combined, such as breathing techniques (e.g., deep‐inspiration breath hold [DIBH]), irradiation techniques (e.g., arc therapy), and radiation modalities (e.g., proton therapy).

The patient position also forms an important characteristic and is less dependent on technological requirements. Most RT centers treat their patients in the supine position, whereas it has been recognized that breast RT in prone position reduces radiation‐induced side effects.^5^, ^6^, ^7^, ^8^ The prone position modifies the target volume by gravity, thus enlarging the distance to the OARs and shortening path lengths through the thorax. The reluctance of hospitals for prone positioning may be caused by inferior comfort, lower setup precision, the inability to target regional lymph nodes, or the absence of specific training for prone positioning.^9^, ^10^, ^11^

In previous studies, we identified two subtypes of prone positioning: prone dive and prone crawl.^12^, ^13^ The prone dive position is mainly used in whole‐breast irradiation (WBI), whereas the prone crawl position was initially designed for targeting WBI and lymph node irradiation (WBI + LNI).^13^ In the latter context, prone crawl proved its ability to lower doses to OARs as compared to supine positioning^14^, ^15^ and improved lymph node access, patient comfort, and setup precision as compared to prone dive positioning.^12^, ^13^

### 1.2 Purpose

It is relevant to also investigate the potential role of prone crawl positioning in WBI‐only, as WBI and its more basic irradiation techniques are more frequent. First, the group of patients requiring WBI‐only is up to three times higher than the group requiring WBI + LNI in countries with early‐detection programs.^16^ Second, RT centers in countries without early‐detection programs are often lacking resources (equipment, technical staff, and so on) to offer advanced irradiation techniques.^17^ As WBI allows for more basic irradiation techniques than WBI + LNI, it thus seems relevant to investigate the effects of different patient positions for basic irradiation techniques. Comparative studies on supine and prone dive positioning for WBI exist, but comparisons with prone crawl positioning are lacking for this context. The first objective of this study is thus to perform a comparison for WBI among three positions (supine, prone dive, and prone crawl) for two basic irradiation techniques (wedged‐tangential fields [W‐TF] and tangential‐field intensity‐modulated RT [TF‐IMRT]) and two more demanding irradiation techniques (multi‐beam IMRT [MB‐IMRT], and intensity‐modulated arc therapy [IMAT]).

Furthermore, the dosimetrical effects of DIBH have been reported for supine and prone dive position in WBI,^18^, ^19^, ^20^ but not for prone crawl WBI. The second objective is thus quantifying the dosimetrical effects of DIBH in the prone crawl position in WBI.

A third objective is to explain the dosimetric differences between the prone dive and prone crawl position by quantifying the anatomical differences.

Altogether, this examination would support hospitals in choosing the proper positioning technique according to their experience and setup. For hospitals without advanced irradiation techniques, in particular, it is relevant to investigate whether a change in patient position could counter the lack of technical resources.

## 2 Methods

Five existing CT‐datasets, referenced as datasets A1 to D, were processed and analyzed (Table 1). In each dataset, new regions‐of‐interest were delineated, and new irradiation techniques were added. Custom treatment planning software was generated, and 252 new treatment plans were made. Together, this specific selection of datasets allows for a comparison of the three patient positions for both basic (W‐TF and TF‐IMRT) and advanced or more time‐consuming (MB‐IMRT and IMAT) irradiation techniques. Table 1 provides an overview of the patient setup, the number of patients, and the irradiation techniques that were studied in each dataset. All patients were involved in clinical studies after written informed consent was obtained for each patient and all studies were approved by the local ethics board.

**TABLE 1:** Overview of patient setup and irradiation techniques for each dataset

Dataset	Patient setup	Number of patients and laterality	Mean age (range) in years	Mean clinical tumor volume (range) in cm^3^	Irradiation techniques				Reference source
A1	Prone dive—supine	6 left‐sided	61 (31–67)	441 (85–1231)	W‐TF	TF‐IMRT	MB‐IMRT	IMAT	Mulliez et al.^24^
A2	Prone dive—supine	6 right‐sided	56 (37–76)	350 (140–503)	W‐TF	TF‐IMRT	MB‐IMRT	IMAT	Mulliez et al.^24^
B	Prone crawl—supine	5 left‐sided	59 (50–72)	482 (195–646)		TF‐IMRT	MB‐IMRT	IMAT	Speleers et al.^14^
C	Prone crawl—prone dive	10 right‐sided	52 (41–59)	486 (109–808)		TF‐IMRT	MB‐IMRT		Deseyne et al.^12^
D	Prone crawl: DIBH–SB	24 left‐sided	54 (41–76)	445 (82–982)		TF‐IMRT		IMAT	Speleers et al.^15^

Abbreviations: DIBH, deep‐inspiration breath hold; IMAT, intensity‐modulated arc therapy; MB‐IMRT, multi‐beam intensity‐modulated radiotherapy; SB, shallow breathing; TF‐IMRT, tangential‐field intensity‐modulated radiotherapy; W‐TF, wedged‐tangential fields.

### 2.1 Patient setup and CT‐simulation

**FIGURE 1:**
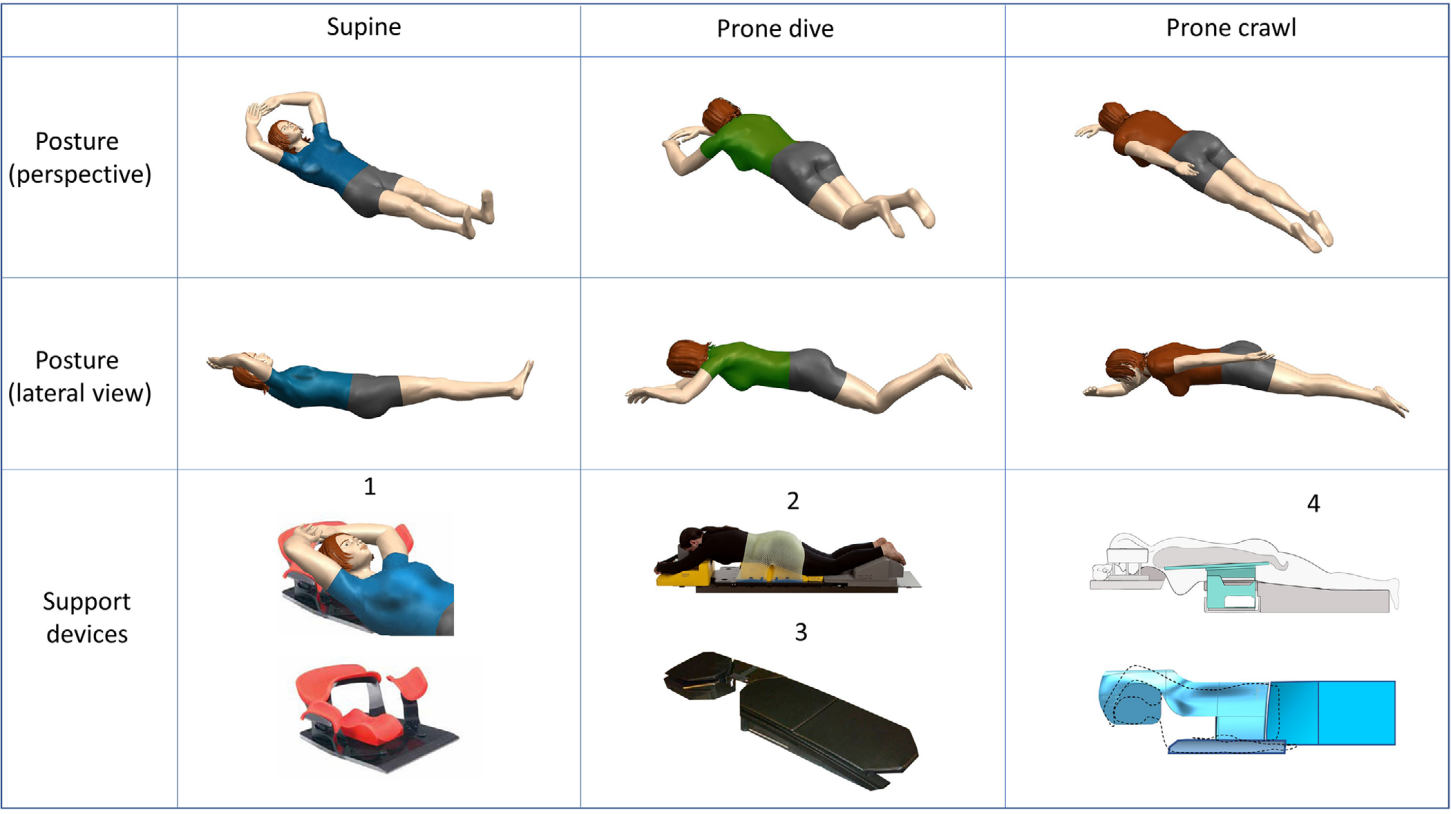
Overview of different patient positioning techniques and the corresponding immobilization devices used in the different datasets. Legend: 1. Posirest (CIVCO Radiotherapy, Orange City, Iowa, USA); 2. modified AIO Prone (Orfit Industries, Wijnegem, Belgium); 3. modified Horizon Prone Breastboard (CIVCO Radiotherapy, Orange City, Iowa, USA); 4. Prone Crawl Breast Couch (Ghent University, Belgium)

Three different patient positions were analyzed: supine, prone dive, and prone crawl. All patients underwent CT‐simulation in two positions, with CT‐slices between 3‐ and 5‐mm thicknesses. Figure 1 illustrates the specific patient positioning techniques and the immobilization devices that were used to accomplish the patient setup.

#### Supine position

Supine positioning (datasets A1, A2, and B) was performed with both arms elevated above the head using the Posirest Arm Support (CIVCO Radiotherapy, Orange City, Iowa, USA). Opaque wires were placed on the borders of each breast to generate the contour of the breast on CT‐images.^21^

##### Prone dive position

Prone dive positioning (datasets A1, A2, and C) was performed with both arms elevated above the head, using a modified Horizon breastboard^9^ (CIVCO Radiotherapy, Orange City, Iowa, USA) in datasets A1 and A2, and a modified AIO‐prone breastboard^22^ (Orfit Industries, Wijnegem, Belgium) in dataset C.

##### Prone crawl position

Prone crawl positioning (datasets B, C, and D) was performed on a Prone Crawl Breast Couch^15^ (Ghent University, Belgium), with the contralateral (CL) arm elevated above the head and the ipsilateral (IL) arm next to the torso.

CT‐images were acquired in shallow breathing (SB) in all datasets and DIBH in dataset D.^15^ The DIBH maneuver of about 15 s was monitored using Respisens magnetic sensors (Nomics, Angleur, Belgium) placed on the surface of the Breast Couch and lateral thoracic wall.^23^

#### 2.1.1 Patient selection

- Datasets A1 and A2

Twelve early‐stage breast cancer patients (six left‐sided and six right‐sided), presenting for WBI after breast conserving surgery, were included in datasets A1 and A2.^24^ New OARs were added in the CT‐dataset of both prone dive and supine plans.

- Dataset B

Five left‐sided breast cancer patients with invasive carcinoma of the breast and pathologically confirmed positive lymph node status were included. All patients underwent lumpectomy and axillary clearance followed by adjuvant WBI + LNI in the supine position.^14^ At the time of the treatment, all patients underwent additional CT‐imaging in the prone crawl position for in silico studies.

- Dataset C

Ten right‐sided patients underwent WBI‐treatment in both the prone dive and prone crawl position.^12^

- Dataset D

Twenty‐four left‐sided patients with invasive carcinoma and pathologically confirmed positive lymph node status were included.^15^

#### 2.1.2 Target and OAR definition

##### Dose prescription and objective

A median dose of 40.05 Gy was prescribed in 15 fractions (2.67 Gy/fraction) for all datasets. The objective was to cover 95% of the target volume with at least 95% of the prescribed dose (i.e., 38.05 Gy), with no more than 5% receiving 107% of the prescribed dose (i.e., 42.85 Gy). All plans were generated for an Elekta Synergy 6‐MV accelerator. The planning target volume (PTV) of the breast (PTV_WBI) was created with a 5‐mm margin around the clinical target volume, except toward the skin surface. For the optimized PTV_WBI (PTV_WBI_opt), a 5‐mm margin around the lungs was subtracted from the PTV_WBI. The contouring guidelines were consistent for all datasets, and the contouring of additional OARs was performed by the two radiation oncologists.

##### Planning software

All plans were made with the GRATIS treatment planning platform (Sherouse Systems Inc., Chapel Hill, NC, USA),^25^ complemented with in‐house developments.^25^, ^26^ Additional custom software developments were implemented to avoid entry dose through the wedge and arm support for IMRT and IMAT‐planning without limiting the gantry angle range. Dose calculations were performed using the convolution–superposition dose computation engine in the Pinnacle 9.8 treatment planning system (Philips Healthcare, Fitchburg, Wisconsin, USA).

##### Treatment planning

In total, 252 new treatment plans were made for this study in the period between November 2020 and July 2021. All plans were drafted by the same treatment planner in order to reduce interobserver variability. Four common irradiation techniques were selected, two basic (W‐TF and TF‐IMRT) and two advanced or more demanding techniques (MB‐IMRT and IMAT).

○ W‐TF

For W‐TF, the positioning of a lead collar around the palpable breast was used to facilitate the definition of the optimal medial gantry angle, the collimator angle, and the field aperture for tangential (wedged and unwedged) photon beams in the virtual simulator. Gantry angles were determined so that the beam aperture contains a minimal lung volume while avoiding the CL breast. Weights of the beams were optimized.

○ TF‐IMRT

TF‐IMRT used the same gantry angles as W‐TF. Field‐in‐field segments were created for dose homogeneity optimization.^26^ Weights of the segments were optimized.^25^

○ MB‐IMRT

MB‐IMRT used a six‐field non‐opposing coplanar beam setup shaped around the PTV_WBI_opt for prone and supine positions. Field‐in‐field segments were created to avoid the IL lung, heart, and CL breast.^26^ Segment apertures and weights were optimized.^25^

○ IMAT

The IMAT plan optimization used a coplanar multiple overlying IMAT technique that exploits optimal beam directions and reduces low‐dose spread to the OARs. Gantry start and stop angles of the arcs resulted from the optimization. IMAT planning tools, developed at Ghent University Hospital as extensions of the GRATIS treatment planning platform are described elsewhere.^27^, ^28^

#### 2.1.3 Dose statistics

For datasets A1, A2, and B with only five or six patients, the standard error of the mean is given in Section 2. For datasets C and D (*n* ≥10), statistical comparison was performed with two‐tailed *t*‐tests, and *p* ≤ 0.05 was considered statistically significant. Additionally, the Wilcoxon signed‐rank test was applied for the OARs of dataset C, as the OAR data had no normal distribution. The dosimetrical range was added in the Supporting Information for all datasets.

##### Dosimetric results

Dose statistics are referred to as *D_n_*: the minimal absolute dose delivered to *n*% of the volume. *D*_02_ and *D*_98_ were used as surrogates for maximum and minimum dose, respectively. These were evaluated for the PTV_WBI_opt, as well as the dose homogeneity defined as

- $\mathrm{Dose}\;\mathrm{homogeneity}:\;1-\left(D_{02}-D_{98}\right)/\left(\mathrm{median}\;\mathrm{dose}\right).$

The following indices were also calculated for the PTV_WBI_opt:

- Jaccardindex:(A∩B)/(A∪B)

where *A* is the volume covered by the PTV_WBI_opt and *B* is the volume covered by the 95% isodose. The Jaccard index increases with an increase in similarity or overlap between the target volume and the 95% isodose and is a measure of dose conformity of the treatment plan

- Dosecoverageindex:(A∩B1)/A

where *B*1 is the volume covered by the 95%–107% isodose, that is, the volume receiving between 38.05 and 42.85 Gy. The dose coverage index calculates the proportion of the target for which the treatment planning objectives are met.

- Mismatchindex:B2/B

where *B*2 is the volume covered by the 95% isodose and lying outside the PTV_WBI_opt. It is the fraction of the ≥95% dose region not overlapping the target optimization ROI. If the mismatch index is large, high amounts of normal tissues receive more than 95% of the prescription dose.

Dose statistics were reported for the following OARs: cardiac structures (heart, LAD, and apex), lung structures (IL lung, CL lung, and lungs combined), and CL breast. In the Supporting Information, dose endpoints for IL arm, esophagus, thyroid, brachial plexus, spinal cord, and lymph nodes (LNN) I–V are added, depending on the relevance to the according dataset. For the IL arm specifically, the point‐maximum dose was evaluated (*D*_max_).

*Dose–volume histograms*. Cumulative dose–volume histograms (DVHs), displaying the simulated radiation dose delivered to a volume of interest, are given for the PTV_WBI_opt, heart, lungs, and CL breast. These DVHs can be found in Supporting Information for all datasets.

#### 2.1.4 Anatomical results

##### Dimensions of CL and IL breast

The average craniocaudal and dorsal–ventral lengths of the IL and CL breast of 10 patients (dataset C) are given in Table 11, for both prone dive and prone crawl positioning. These numbers are reported to anatomically explain dose differences caused by the difference in prone positions.

##### Distance–volume histograms

Cumulative distance–volume histograms (DiVHs) of the PTV to the heart, lungs, and CL breast are given in Figure 3 for datasets C (10 right‐sided patients, prone dive–prone crawl) and D (24 left‐sided patients, prone crawl: SB–DIBH). The DiVHs of datasets A1, A2, and B can be found in Supporting Information as their results are ambiguous and their sample sizes are low for anatomical comparison. The DiVHs display the volume in function of distance in the same way as a DVH depicts the volume in function of the dose. For every point inside a given ROI, the minimal distance to a second ROI is computed. This set of distances is assembled into a cumulative histogram. These data are reported to anatomically explain dose differences caused by different patient positions.

## 3 RESULTS

### 3.1 Dosimetry

#### 3.1.1 Dataset A1: prone dive–supine, left‐sided

Table 2 shows the doses to target structures for datasets A1 and A2. For *D*_98_, *D*_02_, and the dose homogeneity, the most notable difference lies within the lower *D*_98_ and dose homogeneity for IMAT in both positions. This lower conformity is caused by an optimization in favor of the dose to OARs. The Jaccard and Mismatch indices were strongly inferior for the less conformal W‐TF technique (*p* < 0.01) as compared to the other irradiation techniques. Furthermore, the Jaccard and Mismatch indices were inferior (*p* ≤ 0.02) with all techniques for the supine position, with longer path lengths through the irradiated breast. For the dose coverage index, no clinically important differences were found.

**TABLE 2:** Dose–volume parameters and conformity indices for the optimized planning target volume (PTV_WBI_opt) of datasets A1 (left‐sided) and A2 (right sided) combined

	*D*_98_ (Gy)			*D*_02_ (Gy)			Dose homogeneity (%)		
	Prone dive Mean	Supine Mean	*p*‐Value	Prone dive Mean	Supine Mean	*p*‐Value	Prone dive Mean	Supine Mean	*p*‐Value
**W‐TF**	**37.45**	**37.90**	<0.01	**42.22**	**42.79**	0.19	**88.32**	**87.85**	0.75
**TF‐IMRT**	**37.40**	**37.72**	0.01	**41.62**	**42.00**	0.02	**89.51**	**89.51**	0.68
**MB‐IMRT**	**37.43**	**37.48**	0.76	**41.65**	**42.05**	<0.01	**89.50**	**88.62**	0.11
**IMAT**	**37.33**	**36.99**	0.10	**42.49**	**42.18**	0.55	**87.40**	**88.25**	0.23

	Jaccard index (%)			Dose coverage index (%)			Mismatch index (%)		
	Prone dive Mean	Supine Mean	*p*‐Value	Prone dive Mean	Supine Mean	*p*‐Value	Prone dive Mean	Supine Mean	*p*‐Value
**W‐TF**	**69.63**	**43.12**	<0.01	**94.74**	**94.75**	0.23	**18.70**	**46.43**	<0.01
**TF‐IMRT**	**81.58**	**66.15**	<0.01	**95.22**	**96.25**	0.02	**5.50**	**18.94**	0.01
**MB‐IMRT**	**83.00**	**73.38**	<0.01	**95.50**	**96.83**	0.01	**4.08**	**11.33**	<0.01
**IMAT**	**80.86**	**75.75**	0.02	**94.67**	**95.00**	0.75	**5.74**	**8.00**	0.14

*Note*: *p*‐Values for a paired *t*‐test, where *p* < 0.05 is considered statistically significant.

Abbreviations: IMAT, intensity‐modulated arc therapy; MB‐IMRT, multi‐beam intensity‐modulated radiotherapy; TF‐IMRT, tangential‐field intensity‐modulated radiotherapy; W‐TF, wedged‐tangential fields.

Table 3 shows the dose to the OARs of dataset A1, comparing prone dive with supine positioning for left‐sided WBI. For the cardiac structures, mixed results are obtained. The prone dive position shows three‐ to fivefold lower values for the IL lung and integral lung dose with every irradiation technique. For the CL breast, prone dive results in higher doses than supine with all techniques, except for the *D*_02_ of CL breast with W‐TF.

**TABLE 3:** Dose to organs‐at‐risk (OARs) for dataset A1

	W‐TF				TF‐IMRT				MB‐IMRT				IMAT			
	Prone dive		Supine		Prone dive		Supine		Prone dive		Supine		Prone dive		Supine	
	Dose (Gy)	SEM	Dose (Gy)	SEM	Dose (Gy)	SEM	Dose (Gy)	SEM	Dose (Gy)	SEM	Dose (Gy)	SEM	Dose (Gy)	SEM	Dose (Gy)	SEM
Heart (mean)	**1.47**	0.22	**1.16**	0.14	**0.90**	0.07	**0.96**	0.14	**0.96**	0.08	**0.87**	0.16	**1.10**	0.12	**0.89**	0.15
Apex heart (mean)	**12.70**	0.05	**7.51**	1.67	**3.10**	0.33	**4.63**	1.53	**3.52**	0.35	**3.98**	1.31	**4.75**	1.20	**3.90**	1.29
LAD (mean)	**2.70**	0.56	**2.64**	0.37	**1.72**	0.26	**2.39**	0.33	**1.84**	0.26	**1.81**	0.36	**2.03**	0.24	**2.13**	0.52
IL lung (left) (mean)	**0.64**	0.13	**3.19**	0.49	**0.48**	0.06	**2.43**	0.40	**0.49**	0.08	**2.62**	0.31	**0.54**	0.08	**2.06**	0.30
CL lung (right) (mean)	**0.12**	0.01	**0.20**	0.02	**0.11**	0.01	**0.16**	0.02	**0.12**	0.02	**0.15**	0.04	**0.12**	0.02	**0.14**	0.02
Lungs (mean)	**0.34**	0.06	**1.50**	0.23	**0.27**	0.03	**1.15**	0.20	**0.27**	0.04	**1.22**	0.17	**0.29**	0.04	**0.96**	0.14
CL breast (mean)	**0.55**	0.07	**0.45**	0.04	**0.63**	0.08	**0.42**	0.03	**0.65**	0.07	**0.27**	0.04	**0.67**	0.06	**0.38**	0.05
CL breast (*D*_02_)	**1.88**	0.19	**1.99**	0.12	**2.52**	0.66	**1.93**	0.28	**1.99**	0.23	**1.16**	0.12	**2.11**	0.14	**1.79**	0.30

Abbreviations: CL, contralateral; IL, ipsilateral; IMAT, intensity‐modulated arc therapy; MB‐IMRT, multi‐beam intensity‐modulated radiotherapy; TF‐IMRT; tangential‐field intensity‐modulated radiotherapy; W‐TF, wedged‐tangential fields; SEM, standard error of the mean.

#### 3.1.2 Dataset A2: prone dive–supine, right‐sided

(Dose‐to‐target structures are the same as for dataset A1)

Table 4 shows the dose to the OARs of dataset A2, comparing prone dive with supine positioning for right‐sided WBI. For the cardiac structures and CL breast, differences are not clear. We notice that the prone dive position shows two‐ to sevenfold lower values for IL lung and integral lung dose with every irradiation technique. In contrast to its results for left‐sided prone dive position, W‐TF showed competitive to the IMRT and IMAT techniques (note: only for doses to OARs, not for dose‐to‐target). However, in supine position, it proved to be inferior. A minor advantage of the more demanding IMAT can be found with supine, but not with prone dive positioning. In the prone dive position, TF‐IMRT proves to be competitive to the more demanding MB‐IMRT and IMAT techniques, just as for left‐sided WBI.

**TABLE 4:** Dose to organs‐at‐risk (OARs) for dataset A2

	W‐TF				TF‐IMRT				MB‐IMRT				IMAT			
	Prone dive		Supine		Prone dive		Supine		Prone dive		Supine		Prone dive		Supine	
	Dose (Gy)	SEM	Dose (Gy)	SEM	Dose (Gy)	SEM	Dose (Gy)	SEM	Dose (Gy)	SEM	Dose (Gy)	SEM	Dose (Gy)	SEM	Dose (Gy)	SEM
Heart (mean)	**0.42**	0.05	**0.76**	0.04	**0.53**	0.06	**0.64**	0.04	**0.52**	0.05	**0.60**	0.05	**0.51**	0.05	**0.43**	0.02
Heart apex (mean)	**0.26**	0.04	**0.41**	0.05	**0.34**	0.03	**0.34**	0.06	**0.37**	0.05	**0.20**	0.03	**0.37**	0.04	**0.18**	0.02
LAD (mean)	**0.35**	0.05	**0.58**	0.06	**0.51**	0.06	**0.60**	0.06	**0.54**	0.06	**0.55**	0.04	**0.54**	0.06	**0.44**	0.07
CL lung (left) (mean)	**0.09**	0.01	**0.27**	0.03	**0.09**	0.01	**0.22**	0.03	**0.10**	0.01	**0.22**	0.02	**0.10**	0.01	**0.17**	0.04
IL lung (right) (mean)	**1.04**	0.13	**7.79**	1.37	**1.01**	0.09	**5.62**	1.01	**0.78**	0.12	**3.61**	0.48	**0.73**	0.05	**3.01**	0.47
Lungs (mean)	**0.57**	0.09	**4.49**	0.84	**0.56**	0.06	**3.20**	0.58	**0.45**	0.07	**2.09**	0.28	**0.42**	0.04	**1.73**	0.27
CL breast (mean)	**0.50**	0.09	**0.70**	0.11	**0.56**	0.07	**0.69**	0.13	**0.72**	0.07	**0.45**	0.07	**0.70**	0.08	**0.46**	0.06
CL breast (*D*_02_)	**1.81**	0.37	**2.58**	0.30	**1.56**	0.21	**2.91**	0.78	**1.88**	0.23	**1.40**	0.11	**1.85**	0.24	**1.57**	0.15

Abbreviations: CL, contralateral; IL, ipsilateral; IMAT, intensity‐modulated arc therapy; MB‐IMRT, multi‐beam intensity‐modulated radiotherapy; TF‐IMRT; tangential‐field intensity‐modulated radiotherapy; W‐TF, wedged‐tangential fields; SEM, standard error of the mean.

#### 3.1.3 Dataset B: prone crawl–supine, left‐sided

Table 5 shows the dose‐to‐target structures for dataset B. Dose homogeneity was similar for both patient positions within each technique. IMAT resulted in the lowest *D*_98_ and dose homogeneity for both prone crawl and supine positions, caused by an optimization in favor of OARs. The Jaccard Index was superior in prone crawl for all techniques, with similar results for all techniques within both patient positions. The dose coverage index was similar in both patient positions and lowest for IMAT. The mismatch index was lower in prone crawl for all techniques, lowest for prone crawl IMAT (3%), and highest for supine MB‐IMRT (11%), with its longer path lengths through the irradiated breast.

**TABLE 5:** Dose–volume parameters and conformity indices for the optimized planning target volume (PTV_WBI_opt) of dataset B

	*D*_98_ (Gy)				*D*_02_ (Gy)				Dose homogeneity (%)			
	Prone crawl		Supine		Prone crawl		Supine		Prone crawl		Supine	
	Mean	SEM	Mean	SEM	Mean	SEM	Mean	SEM	Mean	SEM	Mean	SEM
**TF‐IMRT**	**37.52**	0.10	**37.22**	0.11	**41.57**	0.27	**41.88**	0.13	**89.89**	0.58	**88.48**	0.33
**MB‐IMRT**	**37.46**	0.16	**37.48**	0.38	**42.80**	0.30	**42.29**	0.07	**86.62**	1.09	**87.97**	0.95
**IMAT**	**36.73**	0.14	**36.86**	0.51	**42.88**	0.26	**42.41**	0.21	**84.89**	0.89	**82.15**	1.61

	Jaccard index (%)				Dose coverage index (%)				Mismatch index (%)			
	Prone crawl		Supine		Prone crawl		Supine		Prone crawl		Supine	
	Mean	SEM	Mean	SEM	Mean	SEM	Mean	SEM	Mean	SEM	Mean	SEM
**TF‐IMRT**	**81.67**	2.23	**72.37**	3.42	**96.04**	0.33	**95.40**	0.42	**7.64**	1.72	**11.15**	1.74
**MB‐IMRT**	**82.43**	1.88	**72.38**	2.83	**93.92**	2.00	**95.93**	0.74	**5.90**	1.33	**12.50**	2.04
**IMAT**	**85.30**	1.44	**73.86**	1.65	**92.87**	1.18	**93.10**	0.74	**2.74**	0.82	**9.86**	2.10

Abbreviations: IMAT, intensity‐modulated arc therapy; MB‐IMRT, multi‐beam intensity‐modulated radiotherapy; TF‐IMRT; tangential‐field intensity‐modulated radiotherapy; SEM, standard error of the mean.

Table 6 shows the dose to the OARs of dataset B, comparing prone crawl with supine positioning for left‐sided WBI. We notice that the prone crawl position causes lower doses to lung structures (two‐ to sevenfold) and—to a lesser extent—to cardiac structures. Doses to CL breast seem similar in both positions for TF‐IMRT and MB‐IMRT but lower for IMAT. For both patient positions, IMAT seems superior to TF‐IMRT and MB‐IMRT.

**TABLE 6:** Dose to organs‐at‐risk (OARs) for dataset B

	TF‐IMRT				MB‐IMRT				IMAT			
	Prone crawl		Supine		Prone crawl		Supine		Prone crawl		Supine	
	Dose (Gy)	SEM	Dose (Gy)	SEM	Dose (Gy)	SEM	Dose (Gy)	SEM	Dose (Gy)	SEM		SEM
Heart (mean)	**2.48**	0.42	**2.52**	0.42	**1.60**	0.20	**2.21**	0.27	**1.51**	0.13	**2.06**	0.30
Heart apex (mean)	**13.43**	3.03	**16.03**	3.78	**8.50**	1.60	**13.53**	2.81	**7.84**	1.70	**12.23**	2.93
LAD (mean)	**8.11**	2.80	**12.73**	0.79	**3.55**	1.01	**11.04**	0.63	**3.76**	1.00	**11.09**	2.52
IL lung (left) (mean)	**0.66**	0.17	**4.70**	0.43	**1.07**	0.27	**4.89**	0.40	**0.73**	0.18	**4.19**	0.46
CL lung (right) (mean)	**0.16**	0.02	**0.23**	0.02	**0.13**	0.02	**0.29**	0.09	**0.12**	0.01	**0.23**	0.02
Lungs (mean)	**0.38**	0.08	**2.14**	0.20	**0.53**	0.13	**2.27**	0.22	**0.39**	0.08	**1.94**	0.23
CL breast (mean)	**0.41**	0.05	**0.35**	0.04	**0.32**	0.05	**0.29**	0.04	**0.29**	0.07	**0.46**	0.08
CL breast (*D*_02_)	**1.46**	0.21	**1.45**	0.11	**1.14**	0.14	**1.21**	0.07	**1.19**	0.29	**2.15**	0.36

Abbreviations: CL, contralateral; IL, ipsilateral; IMAT, intensity‐modulated arc therapy; MB‐IMRT, multi‐beam intensity‐modulated radiotherapy; TF‐IMRT; tangential‐field intensity‐modulated radiotherapy; SEM, standard error of the mean.

#### 3.1.4 Dataset C: prone crawl–prone dive, right‐sided

Table 7 shows the dose‐to‐target structures for dataset C. Dose homogeneity was similar for all observations (*p* ≤ 0.22). The Jaccard index was slightly better in prone crawl (84%) than in prone dive (79%) for TF‐IMRT (*p* = 0.01) and similar in both prone crawl IMRT techniques (*p* = 0.12). For the dose coverage and Mismatch index, no clinically important differences were found.

**TABLE 7:** Dose–volume parameters and conformity indices for the optimized planning target volume (PTV_WBI_opt) of dataset C

	*D*_98_ (Gy) Prone crawl Mean	*D*_02_ (Gy) Prone dive Mean	Dose homogeneity (%) *p*‐Value	Prone crawl Mean	Prone dive Mean	*p*‐Value	Prone crawl Mean	Prone dive Mean	*p*‐Value
**TF‐IMRT**	**37.58**	**37.46**	0.42	**41.70**	**41.93**	0.12	**89.75**	**88.87**	0.22
**MB‐IMRT**	**37.76**			**41.86**			**89.76**		
*p*‐Value	0.07			0.08			0.97		

	Jaccard index (%) Prone crawl Mean	Dose coverage index (%) Prone dive Mean	Mismatch index (%) *p*‐Value	Prone crawl Mean	Prone dive Mean	*p*‐Value	Prone crawl Mean	Prone dive Mean	*p*‐Value
**TF‐IMRT**	**84.33**	**78.78**	0.01	**96.11**	**95.89**	0.37	**4.78**	**5.11**	0.92
**MB‐IMRT**	**85.78**			**96.78**			**3.44**		
*p*‐Value	0.12			0.08			0.08		

*Note*: *p*‐Values for a paired *t*‐test, where *p* ≤ 0.05 is considered statistically significant.

Abbreviations: MB‐IMRT, multi‐beam intensity‐modulated radiotherapy; TF‐IMRT; tangential‐field intensity‐modulated radiotherapy.

Table 8 shows the dose to the OARs of dataset C comparing prone dive with prone crawl positioning for right‐sided WBI. For TF‐IMRT, the prone crawl position resulted in lower doses to the cardiac structures and CL breast for most patients, but the differences were not significant except for LAD (*p* = 0.03). For the lung structures, however, the prone crawl position results in significantly lower doses (*p* = 0.01), foremost to the IL lung. When we compare TF‐IMRT and MB‐IMRT in prone crawl position, we find no clinically important differences.

**TABLE 8:** Dose to organs‐at‐risk (OARs) of dataset C

	TF‐IMRT				
	Prone crawl Dose (Gy)	Prone dive Dose (Gy)	*p*‐Value	MB‐IMRT Prone crawl Dose (Gy)	Prone crawl TF‐IMRT/MB‐IMRT *p*‐Value
Heart (mean)	**0.60**	**0.66**	0.09	**0.46**	0.01
Apex heart (mean)	**0.43**	**0.49**	0.15	**0.37**	0.17
LAD (mean)	**0.46**	**0.59**	0.03	**0.39**	0.02
CL lung (left) (mean)	**0.09**	**0.13**	0.01	**0.08**	0.02
IL lung (right) (mean)	**0.84**	**1.52**	0.01	**0.69**	0.20
Lungs (mean)	**0.49**	**0.82**	0.01	**0.41**	0.18
CL breast (mean)	**0.45**	**0.59**	0.08	**0.35**	0.58
CL breast (*D*_02_)	**2.22**	**1.75**	0.14	**1.30**	0.58

*Note*: Column 4: *p*‐values of a Wilcoxon rank‐sum test, comparing the dose values between prone crawl and prone dive position within TF‐IMRT. Last column: *p*‐values of a Wilcoxon signed‐rank test, comparing the dose values between TF‐IMRT and MB‐IMRT within prone crawl position. For all statistical tests, *p* ≤ 0.05 is considered statistical significance.

Abbreviations: CL, contralateral; IL, ipsilateral; MB‐IMRT, multi‐beam intensity‐modulated radiotherapy; TF‐IMRT; tangential‐field intensity‐modulated radiotherapy.

#### 3.1.5 Dataset D: prone crawl DIBH–SB, left‐sided

Table 9 shows the dose‐to‐target structures for dataset D. With regards to dose homogeneity and *D*_98_, the most notable difference is in the lower values for IMAT, caused by an optimization in favor of OARs. For the Jaccard index, no clinically important differences were found. The dose coverage index was similar for all observations. The mismatch index for TF‐IMRT was significantly better with DIBH (7% vs. 11%, *p* < 0.01), and for IMAT, it was similar in both breathing modalities.

**TABLE 9:** Dose–volume parameters and conformity indices for the optimized planning target volume (PTV_WBI_opt) of dataset D

	*D*_98_ (Gy)			*D*_02_ (Gy)			Dose homogeneity (%)		
	Prone crawl			Prone crawl			Prone crawl		
	DIBH Mean	SB Mean	*p*‐Value	DIBH Mean	SB Mean	*p*‐Value	DIBH Mean	SB Mean	*p*‐Value
**TF‐IMRT**	**37.52**	**37.53**	0.83	**41.96**	**41.56**	<0.01	**88.94**	**89.95**	<0.01
**IMAT**	**37.34**	**37.34**	0.98	**42.23**	**42.14**	0.48	**87.83**	**88.07**	0.52

	Jaccard index (%)			Dose coverage index (%)			Mismatch index (%)		
	Prone crawl			Prone crawl			Prone crawl		
	DIBH	SB		DIBH	SB		DIBH	SB	
	Mean	Mean	*p*‐Value	Mean	Mean	*p*‐Value	Mean	Mean	*p*‐Value
**TF‐IMRT**	**78.47**	**75.31**	<0.01	**95.92**	**95.97**	0.88	**6.99**	**10.71**	<0.01
**IMAT**	**79.68**	**79.51**	0.83	**94.71**	**95.02**	0.36	**5.50**	**5.72**	0.60

*Note*: *p*‐Values for a paired *t*‐test, where *p* ≤ 0.05 is considered statistically significant.

Abbreviations: DIBH, deep‐inspiration breath hold; IMAT, intensity‐modulated arc therapy; SB, shallow breathing; TF‐IMRT, tangential‐field intensity‐modulated radiotherapy.

Table 10 shows the dose to the OARs of dataset D, comparing SB with DIBH for left‐sided WBI in the prone crawl position. We notice that DIBH mostly shows superior (or equal) results to SB for both TF‐IMRT and IMAT. The well‐known cardiac reduction is apparent, with up to threefold dose reductions for apex with both TF‐IMRT and IMAT. The lowest heart doses using DIBH were obtained with IMAT. DIBH results in slight lung dose increases for both TF‐IMRT (*p* = 0.02) and IMAT (*p* < 0.01). For the mean dose to the CL breast, no clinically important differences were observed.

**TABLE 10:** Dose to organs‐at‐risk (OARs) of dataset D

	Prone crawl							
	TF‐IMRT			IMAT				
	DIBH Dose (Gy)	SB Dose (Gy)	*p*‐Value	DIBH Dose (Gy)	SB Dose (Gy)	*p*‐Value	DIBH TF‐IMAT *p*‐Value	SB TF‐IMAT *p*‐Value
Heart (mean)	**1.18**	**1.97**	<0.01	**1.02**	**1.47**	<0.01	<0.01	<0.01
Apex heart (mean)	**4.37**	**13.23**	<0.01	**3.13**	**9.09**	<0.01	0.01	<0.01
LAD (mean)	**4.37**	**7.36**	<0.01	**3.33**	**5.15**	0.01	<0.01	<0.01
IL lung (left) (mean)	**0.74**	**0.66**	0.07	**0.61**	**0.49**	<0.01	<0.01	<0.01
CL lung (right) (mean)	**0.13**	**0.13**	0.88	**0.13**	**0.13**	0.66	0.70	0.31
Lungs (mean)	**0.43**	**0.38**	0.02	**0.36**	**0.30**	<0.01	<0.01	<0.01
CL breast (mean)	**0.48**	**0.50**	0.25	**0.55**	**0.57**	0.47	<0.01	0.01
CL breast (*D*_02_)	**1.53**	**1.56**	0.48	**1.73**	**1.71**	0.94	0.11	0.08

*Note*: Column 4: *p*‐values of a *t*‐test comparing the dose values between DIBH and SB within prone crawl TF‐IMRT. Column 7: *p*‐values of a *t*‐test comparing the dose values between DIBH and SB within prone crawl IMAT. Column 8: *p*‐values of a *t*‐test comparing the dose values between TF‐IMRT and IMAT within prone crawl DIBH. Column 9: *p*‐values of a *t*‐test comparing the dose values between TF‐IMRT and IMAT within prone crawl SB. For all *t*‐tests, *p* ≤ 0.05 is considered statistical significance.

Abbreviations: CL, contralateral; DIBH, deep‐inspiration breath hold; IL, ipsilateral; IMAT, intensity‐modulated arc therapy; SB, shallow breathing; TF‐IMRT, tangential‐field intensity‐modulated radiotherapy.

### 3.2 Anatomy

#### 3.2.1 Dimensions of IL and CL breast

**FIGURE 2:**
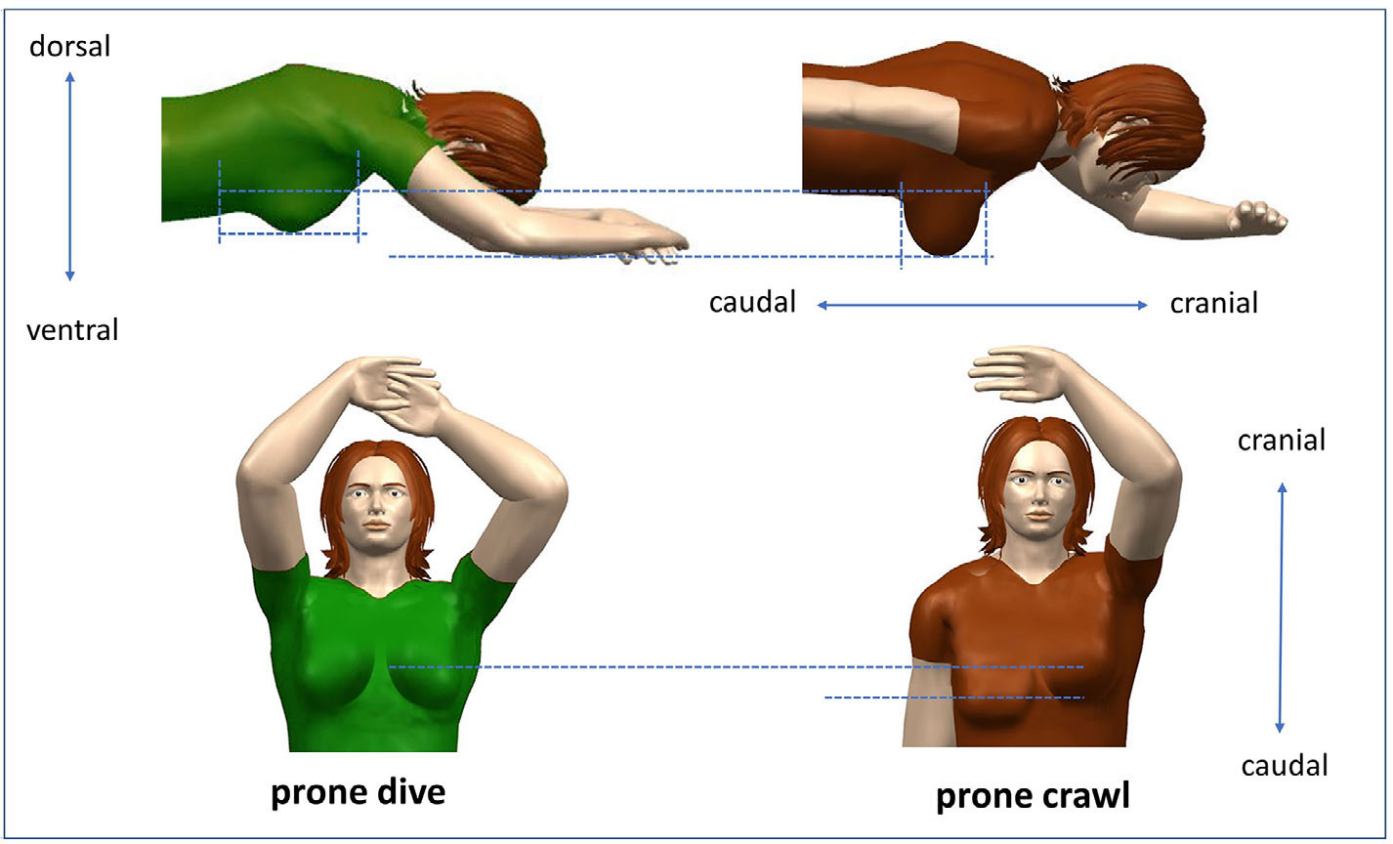
Visualization of anatomical differences between prone dive and prone crawl position. The images on the left represent the prone dive position, the images on the right represent the prone crawl position. The differences are portrayed for the dorsal–ventral direction and the craniocaudal direction, based on the outcomes of Table 11.

In Table 11, the craniocaudal and dorsal–ventral sizes of the IL and CL breast are given for both prone dive and prone crawl positioning (dataset C, 10 right‐sided patients). Figure 2 is a visual representation of the outcomes of Table 11. As for the IL breast, results show that there are significant differences between prone dive and prone crawl position, in both craniocaudal (*p* < 0.01) and dorsal–ventral direction (*p* = 0.05). In prone crawl position, the IL breast gets stretched out less in craniocaudal direction and sags deeper than in prone dive position. As for the CL breast, no significant difference was found between prone dive and prone crawl for the craniocaudal dimension (*p* = 0.12). This could be expected, as the position of the CL arm is similar in prone dive and prone crawl. When we look at the prone dive position (both arms elevated), we do not find significant craniocaudal differences (*p* = 0.92) between the CL and IL breasts, again self‐evident. For the prone crawl position (IL arm alongside the torso and CL arm elevated), however, we find a significant craniocaudal difference between both breasts (*p* < 0.01). In Figure 2, the anatomical differences between prone dive and prone crawl are portrayed, with a focus on the craniocaudal shift and the further sagging of the IL breast in prone crawl position. The results of Table 11 are expected to be similar for left‐sided patients as the position is a symmetric copy. However, the distances to OARs of the PTV may be different, as organs such as the heart are not in a symmetrical position.

**TABLE 11:** Dimensions of ipsi‐ and contralateral breast

	Structure				
		Ipsilateral breast		Contralateral breast	
Direction	Craniocaudal length (cm)	Prone dive	Prone crawl	Prone dive	Prone crawl
		15.35	11	15.4	14.75
			***p*‐Value**		***p*‐Value**
			<0.01		0.12
	**Dorsal–ventral length (cm)**	**Prone dive**	**Prone crawl**		
		9.8	10.8		
			***p*‐Value**		
			0.01		

	Patient position				
		Prone dive		Prone crawl	
Direction	Craniocaudal				
Length (cm)	Ipsilateral breast	Contralateral breast	Ipsilateral breast	Contralateral breast	
		15.35	15.40	11	14.75
			***p*‐Value**		***p*‐Value**
			0.92		<0.01

*Note*: In these charts, the average lengths in both craniocaudal and dorsal–ventral direction are given for the prone dive and prone crawl position. The sample consists of 10 patients (dataset C, right‐sided WBI) and is the same for both positions. The upper chart compares the average dimensions between prone dive and prone crawl within the same breast (left panel: IL breast, right panel: CL breast). The lower chart compares the average dimensions between the IL and CL breast within the same patient position (left panel: prone dive, right panel: prone crawl). The *p*‐values are computed using a two‐tailed *t*‐test, where *p* < 0.05 is considered statistically significant.

Abbreviation: CL, contralateral; IL, ipsilateral; WBI, whole‐breast irradiation.

#### 3.2.2 Distance–volume histograms

**FIGURE 3:**
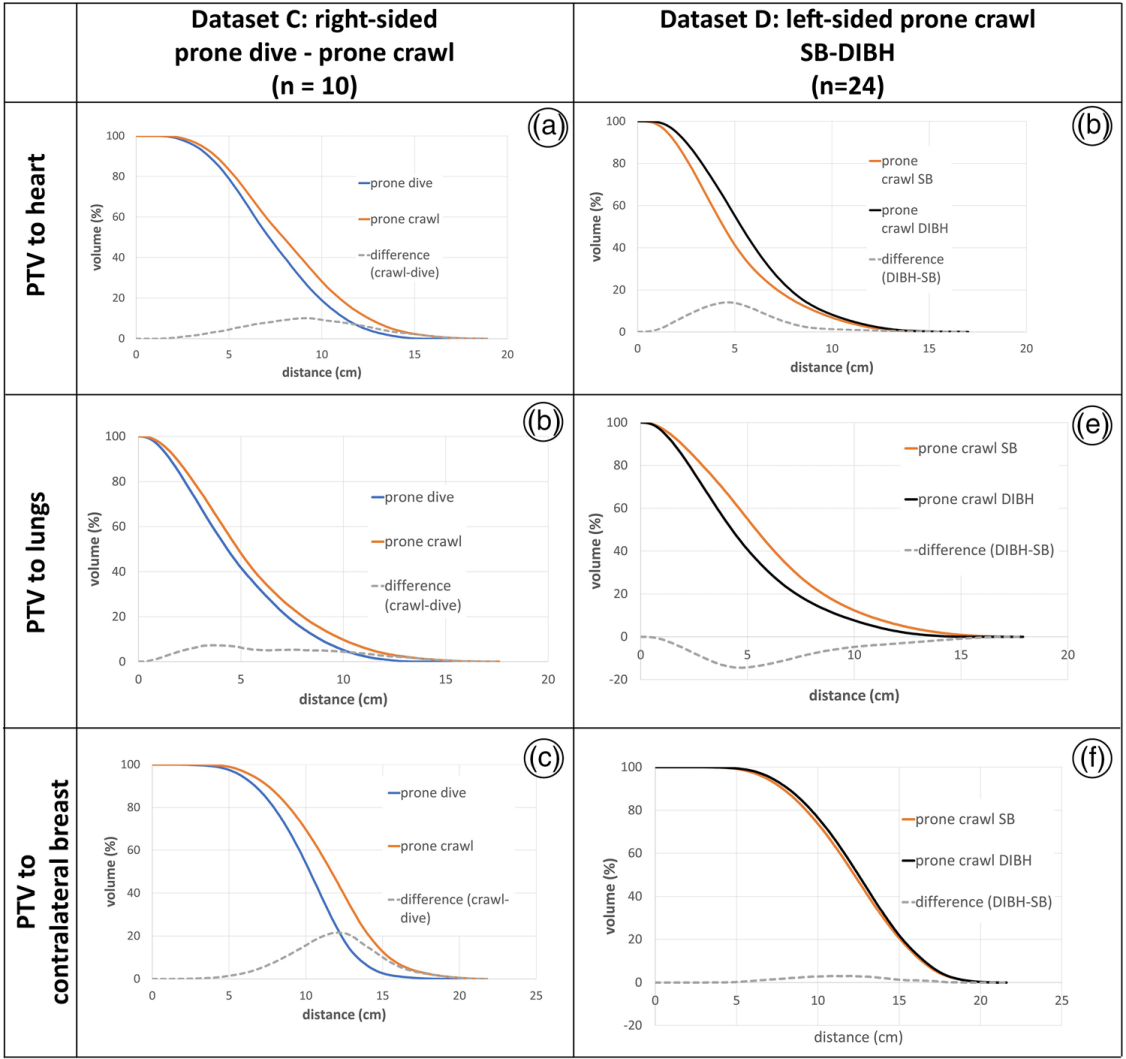
Distance–volume histograms (DiVHs) of planning target volume (PTV) to organs‐at‐risk (OARs). The second column (panels a–c) shows the DiVHs for 10 right‐sided patients of dataset C, in both prone crawl and prone dive position. The third column (panels d–f) shows the DiVHs for 24 left‐sided patients of dataset C, both in prone crawl position, with deep‐inspiration breath hold (DIBH) or shallow breathing (SB). The second, third, and fourth rows represent the DiVHs for the heart, lungs, and contralateral (CL) breast, respectively. The dotted line in each panel indicates the volumetric difference between the examined lines.

Figure 3 displays the cumulative DiVHs of PTV to the heart, lungs, and CL breast, for both datasets C and D. For dataset C (10 right‐sided patients, prone dive–prone crawl), the results show that the PTV is, on average, unambiguously further away from each of the three critical organs in prone crawl than in prone dive position. This larger distance enables lowering the radiation exposure of these organs. In Table 9, we find small yet significant dosimetrical differences. For dataset D (24 left‐sided patients, prone crawl: SB–DIBH), the results are mixed. With DIBH, the distance of the PTV to heart is larger than with SB as the heart retracts, thus enabling the examined heart dose reduction (Table 10). However, the distance of the PTV to the lungs is smaller with DIBH, as the enlarged lung volume replaces parts of the retracted heart. (The effect of the enlarged lung volume at large distances from the PTV is not included in this graph.) In Table 10, we find a slightly yet significantly higher dose to lungs for DIBH as compared to SB. Finally, the distance of the PTV to the CL breast is only slightly smaller with DIBH. This can be expected, as the prone crawl positioning platform fixates both the CL and IL breast. The respiratory movement of the DIBH is mostly performed through the dorsal part of the torso. In Table 10, we also do not find significant differences for the dose to CL breast between DIBH and SB.

## 4 DISCUSSION

We can now discuss the reported dosimetrical differences by evaluating the influence of the patient position and the irradiation technique. Based on the results, we can also take a closer look at the simple prone techniques and whether they can overcome the sophisticated supine IMAT benchmark. This would allow hospitals to achieve better results with low‐tech changes. Lastly, we evaluate our results through a literature study.

### 4.1 Explaining dosimetrical differences: patient position

The dosimetrical differences in the examined datasets can partly be explained by the anatomic variations in the different patient positions. First, there were no distinctive dose differences for the heart between the examined positions, as also concluded in other studies.^29^, ^30^ However, we found that left‐sided prone crawl positioning lowers cardiac doses as compared to supine positioning (dataset B, Table 6), and that right‐sided prone crawl positioning significantly lowers LAD doses as compared to prone dive positioning. For 8 out of 10 patients, the heart dose was lower with prone crawl positioning, although no significance was reached (dataset C, Table 8). Again, these differences are presumably caused by the larger distance of the heart to the irradiated breast in the prone crawl position. Nevertheless, the heart dose reduction through prone crawl positioning is relatively small compared to its lung‐sparing capacity and the heart dose reduction rates of DIBH in all patient positions. Thus, for scenarios with an emphasis on heart sparing, the DIBH technique remains most effective. In dataset D (Table 10), we have found that using DIBH in prone crawl position significantly lowers cardiac doses (up to threefold for apex), at the expense of slightly higher lung doses, as compared to SB. This can be explained by the enlarged lung volume in DIBH, which occupies the space closer to the irradiated breast that is left by the medially and caudally retracting heart. This issue may be resolved by deploying noncoplanar instead of coplanar beams.^15^

Second, for the lungs, we found the well‐known dosimetrical benefit of deploying prone as compared to supine positioning. Lung dose reductions were up to five‐ and sevenfold for left‐sided prone dive and crawl, respectively. Moreover, we found that prone crawl is significantly superior to prone dive positioning for lung structures with right‐sided WBI‐patients (dataset C, Table 8). We presume this is caused by the larger distance between the PTV and lungs in the prone crawl position, as the irradiated breast sags deeper (Figure 2).

Finally, for the CL breast, the results were mixed, with doses and dose differences that were relatively small in all datasets. In cases of superiority for supine position, the greater proximity of the CL breast to the irradiated breast in prone positions may explain this disadvantage. Then again, compared to prone dive, the prone crawl position may suffer slightly less from this drawback (dataset C, Table 8), as there can be a craniocaudal shift between the IL and CL breast, caused by the different arm positions (Figure 2). Furthermore, the irradiated breast is also likely to sag less in prone dive position (Table 11, Figure 2). Because of this larger craniocaudal stretch in prone dive position, the size of the PTV_WBI for this direction is bigger than in prone crawl position, thus causing higher doses to the CL breast. In dataset C (Table 8), the mean dose to CL breast was reduced with prone crawl positioning for 9 out of 10 patients, but no significance was reached.

### 4.2 Explaining dosimetrical differences: irradiation techniques

For W‐TF, we found that there is a remarkable difference for cardiac doses between left‐ and right‐sided WBI in prone dive. In left‐sided WBI (dataset A1, Table 3), W‐TF is inferior for cardiac structures compared to other prone dive techniques. With the heart relatively close to the target volume, this less conformal technique cannot overcome the benefits of IMRT and IMAT. In right‐sided WBI, however (dataset A2, Table 4), W‐TF seems a competitive prone dive technique for cardiac structures (notwithstanding its inferior target results). Presumably, this is because the heart is further away from the target and the IMRT techniques cause more scatter dose to the heart than W‐TF. IMRT techniques can control dose homogeneity to the target, unlike W‐TF, and the IMRT techniques exploit this possibility to create significantly better Jaccard and mismatch indices, at the expense of somewhat higher dose to the heart. Finally, IMAT seems especially effective in supine as this position enables its capability for targeting curved structures like the IL breast. However, this benefit is not big enough to overcome the lung‐sparing capacities of both prone positions.

### 4.3 Simple prone techniques versus the advanced supine IMAT

For RT centers without access to advanced techniques like IMAT, we can investigate whether a different patient position can counter the lack of technical resources. We do this by investigating whether “basic” prone techniques (W‐TF, TF‐IMRT, and MB‐IMRT) may compete with the sophisticated supine IMAT technique, for both left‐ and right‐sided patients. For left‐sided patients, we find that prone dive may be competitive to supine IMAT with TF‐IMRT and MB‐IMRT, but not with the inferior W‐TF (dataset A1, Table 3). The three‐ to fivefold lung dose reduction of prone dive TF‐IMRT and MB‐IMRT is notable, whereas the differences for cardiac structures and CL breast are relatively small. With prone crawl positioning, we also find a remarkable lung dose reduction with both TF‐IMRT and MB‐IMRT, and minor benefits, for the CL breast as compared to supine IMAT (dataset B, Table 6). However, prone crawl TF‐IMRT struggles with relatively higher cardiac doses, whereas prone crawl MB‐IMRT surpasses supine IMAT for all structures. For right‐sided patients, we find that prone dive may again be competitive to supine IMAT with TF‐IMRT and MB‐IMRT, and even with W‐TF (dataset A2, Table 4). However, this should be nuanced, as prone dive W‐TF shows inferior target results. For prone crawl, a direct comparison with right‐sided supine IMAT is lacking, but in dataset C, we compared prone crawl with prone dive. Here, the dose to the LAD and lung structures was significantly lowered with both prone crawl TF‐IMRT and MB‐IMRT. The cardiac advantage for prone crawl is minor.

### 4.4 In comparison

With advances in cancer diagnosis and treatments, survival rates of breast cancer patients are continuously increasing. This implies that a growing number of patients may live to see the late effects of their cancer treatment. For patients with cardiac or pulmonary risk factors, adjuvant RT may even be detrimental.^3^, ^31^ Hence, strategies for limiting doses to OARs whilst maintaining adequate target coverage are essential. The patient position has therefore been investigated in different settings throughout literature.

Breast radiation therapy may cause cardiac injury, such as myocardial infarction and ischemic heart disease,^1^, ^2^, ^32^ with highest risks for patients with left‐sided breast cancer. Cardiac morbidity generally appears 5–10 years after radiation exposure of the heart. Darby et al. reported a linear increased risk of 7.4% for cardiac injury for each Gy increase in mean heart dose, without an apparent threshold.^3^ To limit cardiac exposure, there is no general consensus on the added value of prone dive compared to supine positioning. Saini et al.^30^ found some studies to report heart dose reductions with prone dive positioning for right‐sided patients, large‐breasted patients, and a majority of small‐breasted patients,^5^, ^7^ whereas other studies only found this to be true for patients with large breast volumes.^6^, ^33^, ^34^ The third group of studies reports that the prone dive position may cause a heart dose increase with some patients, because of the proximity to the treated area.^35^ In our study, most differences in heart dose were not decisive, and we could not find significant correlations of breast size to the reported doses. However, we should point out the dose reductions for apex and LAD with a left‐sided prone crawl, as compared to supine position (dataset B, Table 6). This is similar to the outcomes of previous WBI + LNI studies on prone crawl and supine positioning (with IMAT‐planning). Here, no significant differences for the heart were found, yet the LAD dose was largely reduced with the prone crawl setup.^11^, ^14^

To enable heart sparing, there is more consensus on the added value of breathing modalities.^29^, ^36^ Techniques such as DIBH are known to be beneficial for WBI, in both prone dive^19^, ^20^, ^30^ and supine^29^, ^36^, ^37^ positions. In a recent meta‐analysis, Lai et al. found that the combination of DIBH and prone dive positioning achieved superior heart (and lung doses) as compared to supine DIBH and prone dive SB.^19^ This was also confirmed in a factorial design study by Saini et al.^20^ In our current study, the DIBH‐maneuver also induced significant dose reductions to all cardiac structures in the prone crawl position (dataset D, Table 10). This trend is similar to the outcomes of earlier WBI + LNI studies on the effects of DIBH in the prone crawl position, with a 40% heart dose reduction compared to SB.^15^ Previous studies at our center further confirmed the reproducibility of DIBH for both prone dive (WBI)^38^ and prone crawl (WBI + LNI).^39^

Irradiation of lung structures is associated with lung fibrosis, pneumonitis, and secondary lung cancers.^40^ These acute and late side effects are particularly important for early‐stage and young patients who have a high probability of long‐term breast cancer survival,^4^ and for patients with pulmonary risk factors.^31^, ^41^ To enable lung sparing, the added value of the prone dive position is widely acknowledged.^4^, ^19^, ^20^, ^30^, ^37^, ^40^, ^42^ Significant lung sparing was also attributed to the prone crawl position in WBI + LNI studies, with mean lung dose reductions of up to 50% compared to supine positioning.^11^, ^14^ For long‐term smokers, the prone crawl position in WBI + LNI would thus reduce the absolute risk of secondary lung cancer with 2.7%, a 50% risk reduction compared to supine position.^14^ The current study results are in accordance with earlier findings: with both prone positions an important dosimetrical benefit for the lungs can be obtained, as compared to supine position (datasets A1, A2, and B; Tables 3, 4, and 6). Additionally, we found that the largest lung advantages were achieved with the prone crawl position (dataset C, Table 8). Changing the patient position to prone crawl in WBI could thus further lower excess mortalities due to secondary lung cancer.

Irradiation of the CL breast may lead to skin toxicity and secondary breast cancer. With modern techniques, doses to the CL breast are rather small. Taylor et al. estimated that the absolute risk of CL breast cancer from modern RT should be under 1%, and the risk of death from this late effect should be even smaller.^31^ Dose reductions remain nevertheless important, foremost to a young patient population.^43^ To spare the CL breast, literature shows no consensus on the added value of prone dive as compared to supine positioning. The reported differences and absolute doses (<1 Gy) are small,^44^ and most studies show similar results for the CL breast with prone dive and supine position.^33^, ^45^, ^46^ These outcomes are obviously subjected to the positioning of the CL breast and the geometry of the positioning devices. In our current study, the outcomes were not decisive either, with mixed results and small differences (<1 Gy).

In the Supporting Information, we reported doses to the IL arm for all prone crawl setups. In a study on 183 WBI + LNI patients, RT‐induced toxicity to the arm was associated with dose to the brachial plexus and dose to a volume, including the humeral head, the processus coracoideus, the acromion, and the acromioclavicular joint,^47^ rather than with dose to the arm itself. Johansen et al. found a 15‐Gy threshold (for the humeral region) that correlates with arm stiffness, arm pain, use of arm, and shoulder abduction difference. In our study, the average maximum dose of the IL arm for all examined prone crawl setups was 6.4 Gy, with a median of 3.1 Gy. The average and median *V*_15 Gy_ were 0.01 cm^3^. These are below the 15‐Gy threshold, which is to be expected as WBI‐only patients are less exposed to irradiation in this region.

Finally, it is relevant to further compare our results with those of a similar WBI study at our center. Mulliez et al. compared the prone dive and supine position for W‐TF, TF‐IMRT, and MB‐IMRT, without including IMAT, DIBH, or prone crawl position.^24^ Here, it was found that right‐sided prone dive IMRT techniques are superior to supine W‐TF and IMRT. Our study confirms these findings and further adds the competitiveness of right‐sided prone dive IMRT to supine IMAT. Furthermore, Mulliez et al. found that dosimetrical differences between TF‐IMRT and MB‐IMRT are minor in the prone dive position. This is relevant as MB‐IMRT is more time‐consuming in treatment planning and beam delivery. Our study confirms this finding (datasets A1 and A2, Tables 3 and 4), and further brings an important difference with the prone crawl position. The cardiac advantages of prone crawl MB‐IMRT, compared to TF‐IMRT, are notable (dataset B, Table 6). The differences between TF‐IMRT and MB‐IMRT are larger for the prone crawl than for the prone dive position in left‐sided WBI.

### 4.5 Limitations

This is the first study to evaluate the prone crawl position in the WBI‐context. Furthermore, it is the first study to evaluate the three examined positioning techniques for both basic and advanced irradiation techniques. Finally, this article is the first to explain the dosimetrical differences between the prone dive and prone crawl position by quantifying anatomical differences. This comparison could support hospitals in choosing the proper technique according to their experience and setup. Nevertheless, it remains important to discuss the limitations of this study.

A first limitation is the small sample size in datasets A1, A2, and B. This prevented us from performing statistical testing on these data. Furthermore, we could not investigate the role of patient‐specific parameters such as breast size due to these low sample sizes. In these datasets, we worked with CT‐data from previous studies, in which patients were scanned in two patient positions. These data are valuable, as we are not able to retake such studies on a bigger patient population today. With the ALARA principle in mind, we could not pass an ethical committee for larger sample sizes with the prone dive or supine position. A second limitation is the incomplete direct comparison for all patient positions and irradiation techniques. As for the patient position, we lack a direct comparison between the prone crawl and prone dive position for left‐sided patients, and between prone crawl and supine positioning for right‐sided patients. As we worked with existing CT‐datasets and could not perform new trials, we were bound by these limitations. As for the irradiation techniques, we made a trade‐off on the added value of extra techniques. Some techniques (e.g., W‐TF in datasets B–D) were left out, as their relevance was not high in comparison with the other techniques in that dataset.

## 5 CONCLUSION

The target coverage was inferior for W‐TF and supine position. W‐TF proved to be overall inferior, and IMAT proved to be foremost effective in the supine position. TF‐IMRT showed competitive to the more demanding MB‐IMRT and IMAT techniques in the prone dive, but not in the prone crawl position. The important lung‐sparing effect was confirmed for both prone dive and prone crawl positioning and was largest for prone crawl. For the heart, no differences were found between prone dive and supine, whereas the left‐sided prone crawl position showed cardiac advantages, although minor compared to the established heart‐sparing effect of DIBH. Dose differences for CL breast were minor between the patient positions. Compared to prone dive, advantages for prone crawl positioning can be explained by a larger distance between the target volume and OARs. Altogether, the prone dive and prone crawl position proved to be valid alternatives to the supine position in WBI, with largest advantages for the lung structures. For the heart (and CL breast), differences are small, which establishes the role of DIBH in different patient positions. The outcomes of this study may be of particular interest to radiotherapy centers with limited technical resources, for which a switch in patient setup may counter the lack of advanced irradiation techniques.

## CONFLICT OF INTEREST

Ghent University owns the patent application entitled Radiotherapy Board and Couch [WO2015144654A1] filed on March 25, 2014 for which Wilfried De Neve, Bruno Speleers, and Liv Veldeman are listed as inventors.

## AUTHOR CONTRIBUTIONS

Bruno Speleers, Werner De Gersem, Jan Detand, and Wilfried De Neve participated in the design and coordination of the study and helped to draft the manuscript. Max Schoepen and Bruno Speleers conceived of the study, participated in the design and coordination of the study, participated in the treatment planning, carried out the dose calculations, performed the statistical analysis, and drafted the manuscript. Vincent Vakaet and Pieter Deseyne added organs‐at‐risk delineations in the patient planning files and performed consensus reviewing of all targets and organs‐at‐risk. Liv Veldeman and Wilfried De Neve supervised all studies, in which Annick Van Greveling performed patient positioning and CT‐simulation with all patients. Leen Paelinck converted the data of the treatment planning software for statistical analysis. All authors read, corrected, and approved the final manuscript.

## ETHICS STATEMENT

All patients were involved in clinical studies after written informed consent was obtained for each patient and all studies were approved by the local ethics board. Every aspect was performed in‐line with the 1964 declaration of Helsinki and all subsequent revisions. No trial registration was performed because there were no therapeutic interventions.

## Supporting information

FigureS01

FigureS02

TableS01

## Data Availability

All data generated or analyzed during this study are included in this published article (and its Supplementary Information files).
