# Supplementary material for: Four irradiation and three positioning techniques for whole‐breast radiotherapy: Is sophisticated always better?
Source: J Appl Clin Med Phys. 2022 Sep 15;23(11):e13720. doi: 10.1002/acm2.13720 (PMC9680580; doi:10.1002/acm2.13720)

**Addendum**

**Dose-volume histograms**

Cumulative dose-volume histograms (DVHs), displaying the simulated radiation dose delivered to a volume of interest, are given for: PTV_WBI_opt, heart, lungs and CL breast. The DVHs are grouped per dataset.


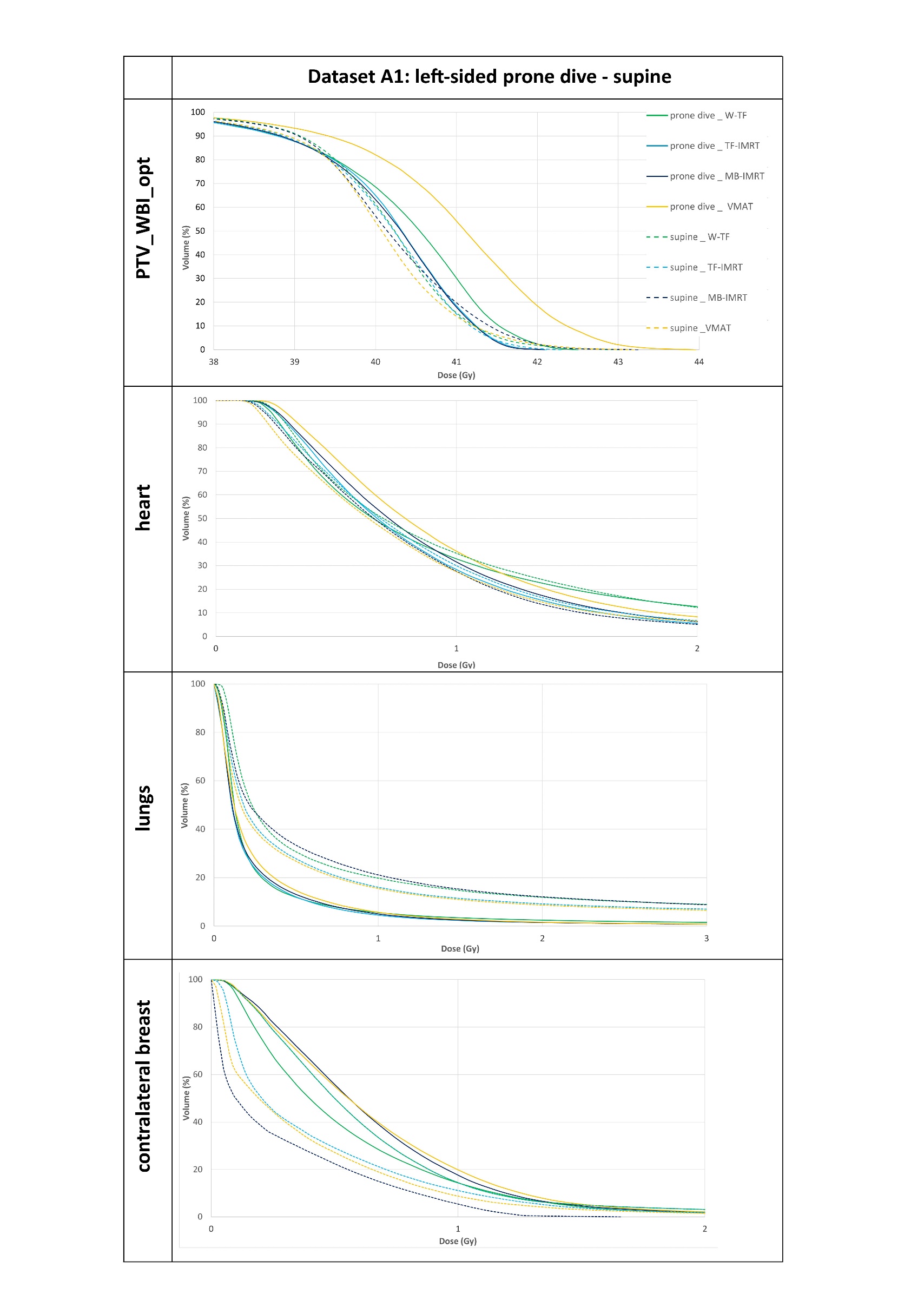

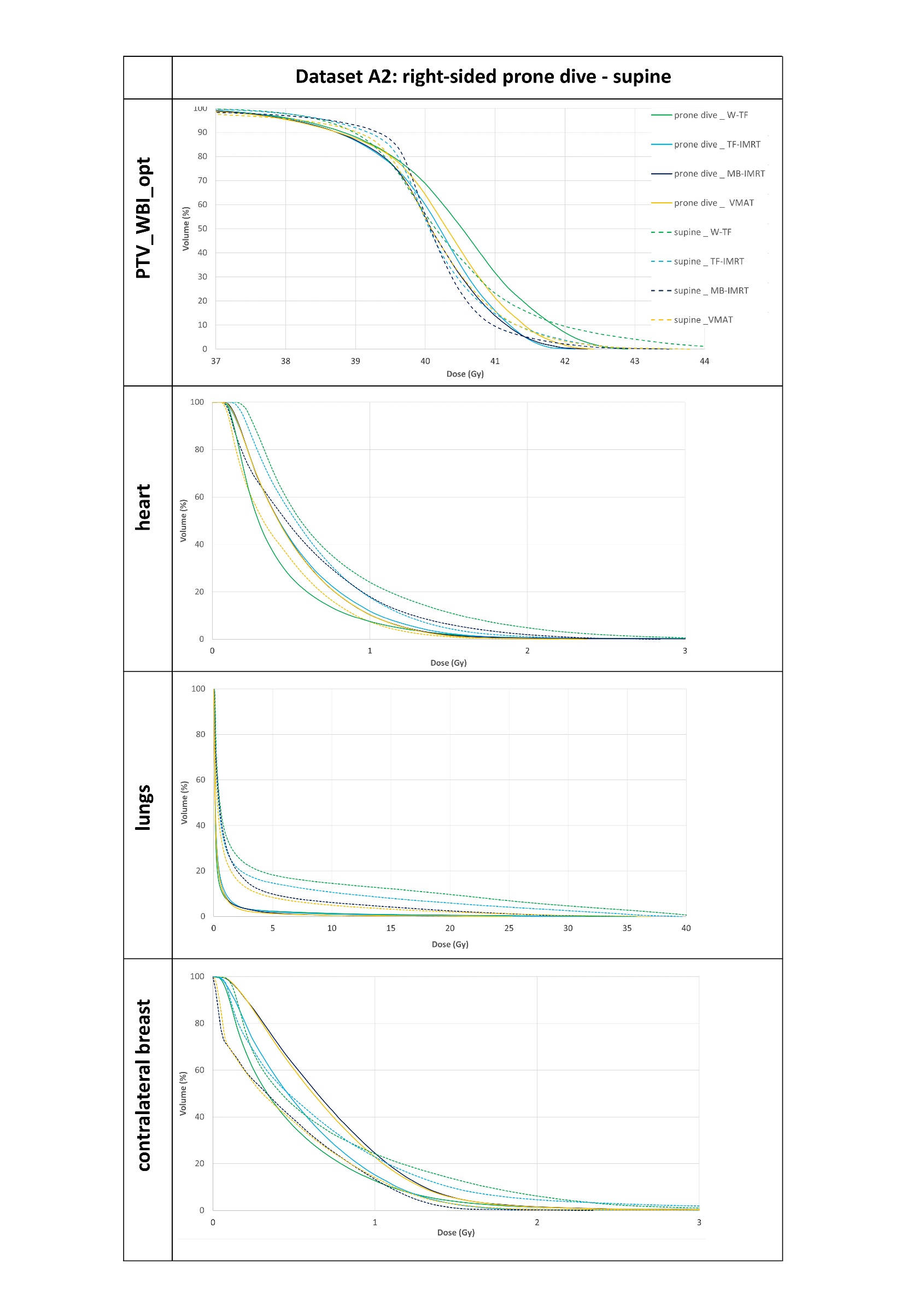


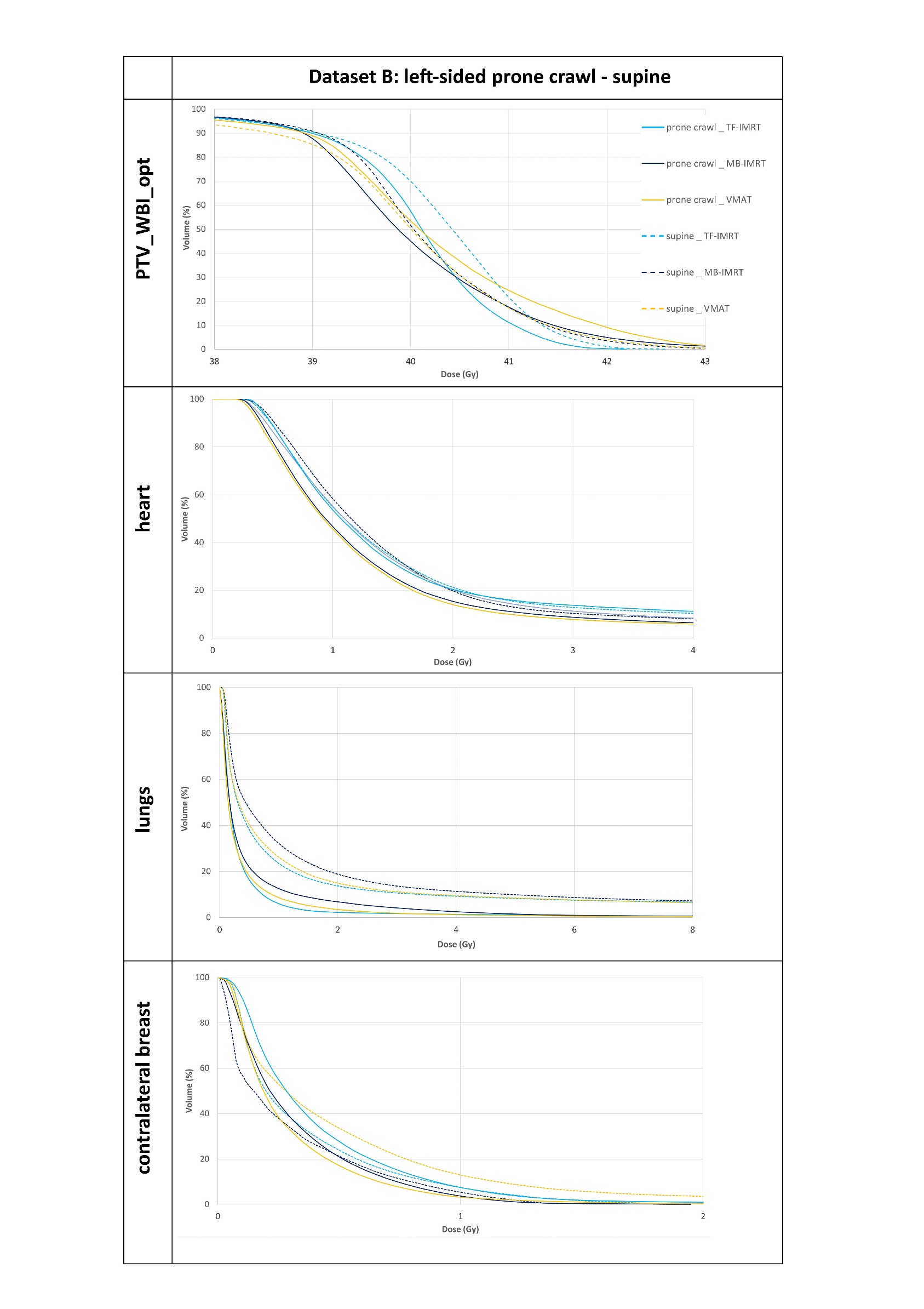


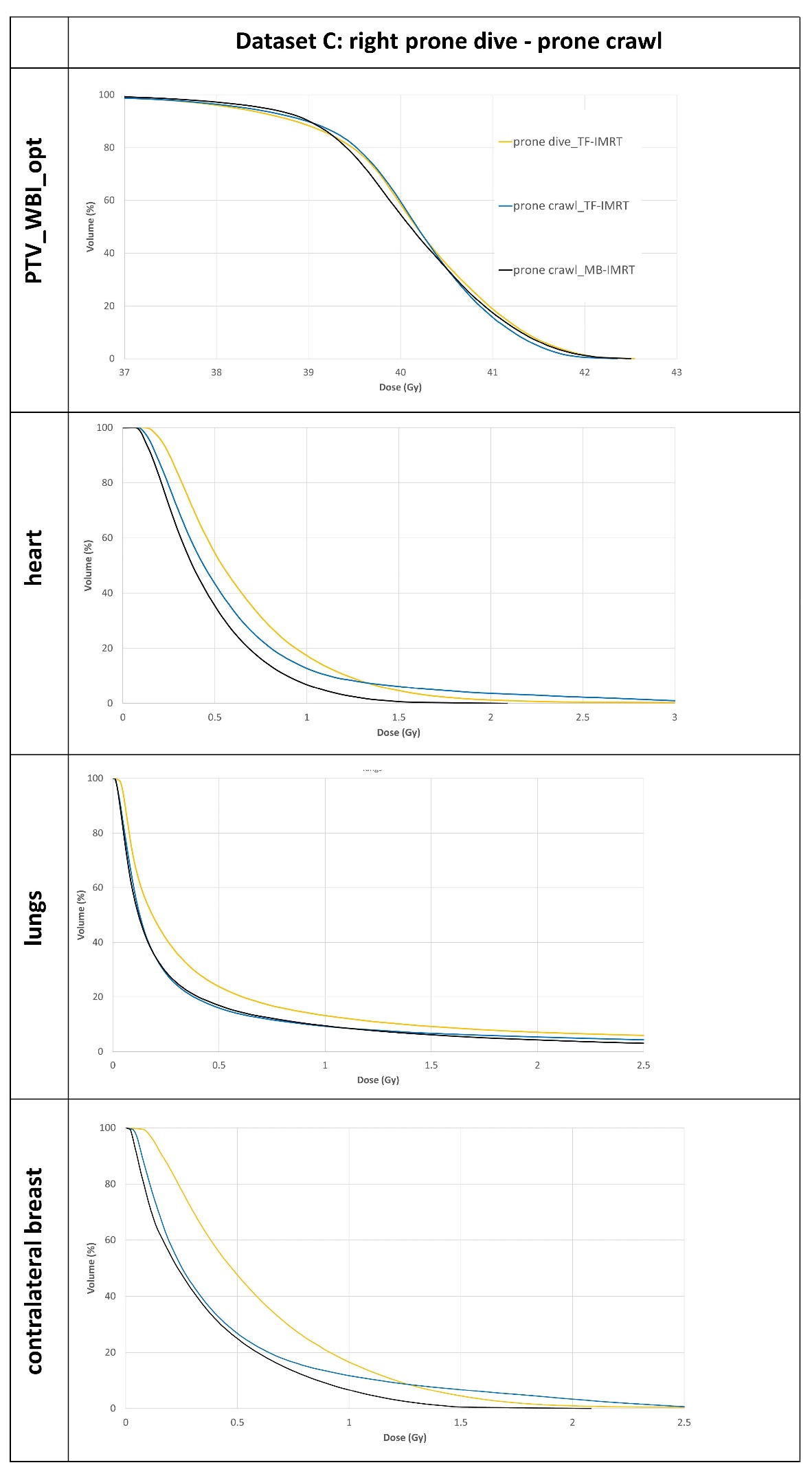


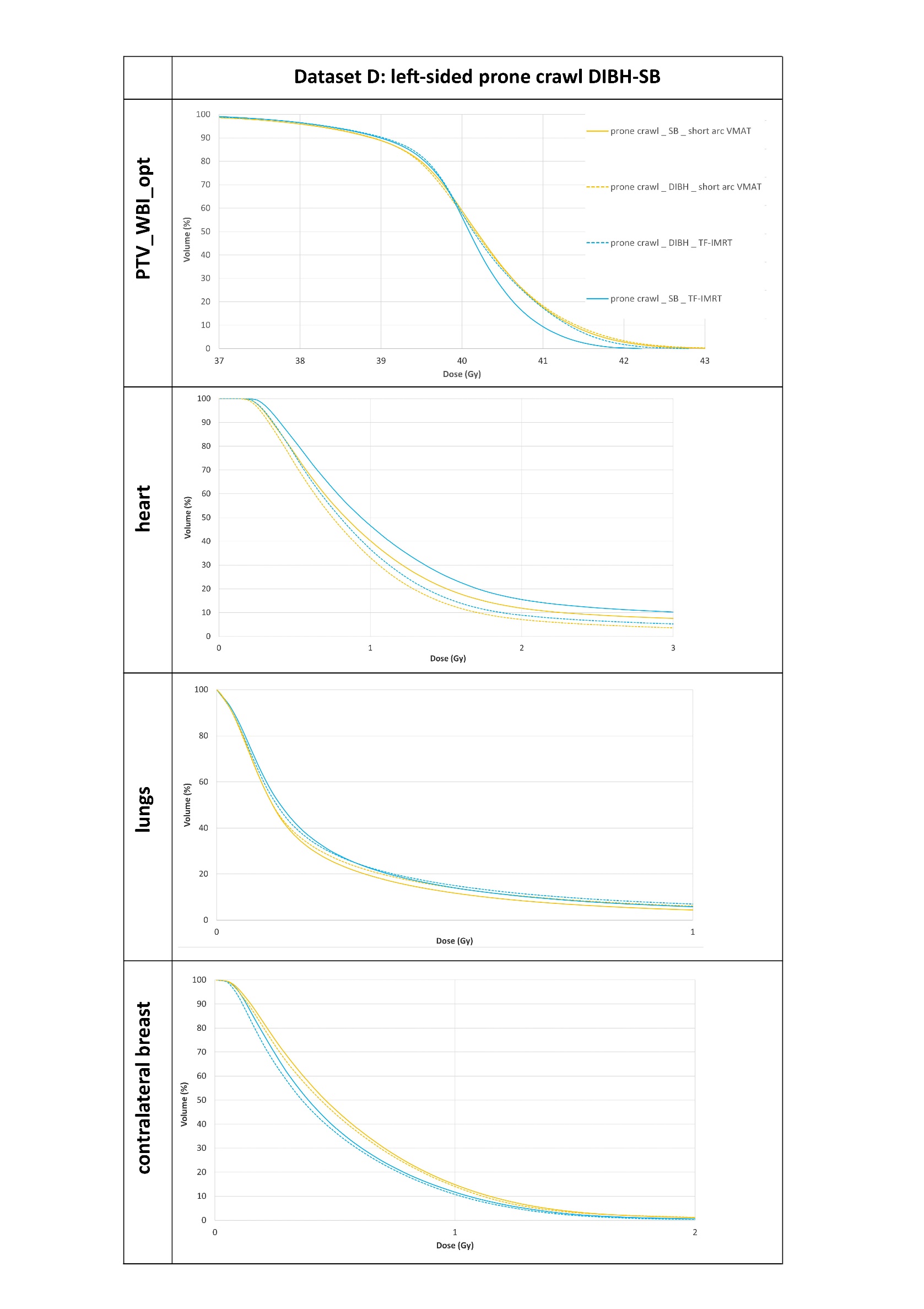

Supplement: Supplementary file 1 — FigureS01 [file ACM2-23-e13720-s001.doc]
