# Supplementary figures and images for: Four irradiation and three positioning techniques for whole‐breast radiotherapy: Is sophisticated always better?
Source: J Appl Clin Med Phys. 2022 Sep 15;23(11):e13720. doi: 10.1002/acm2.13720 (PMC9680580; doi:10.1002/acm2.13720)

**Distance-Volume histograms: datasets A1, A2 and B**


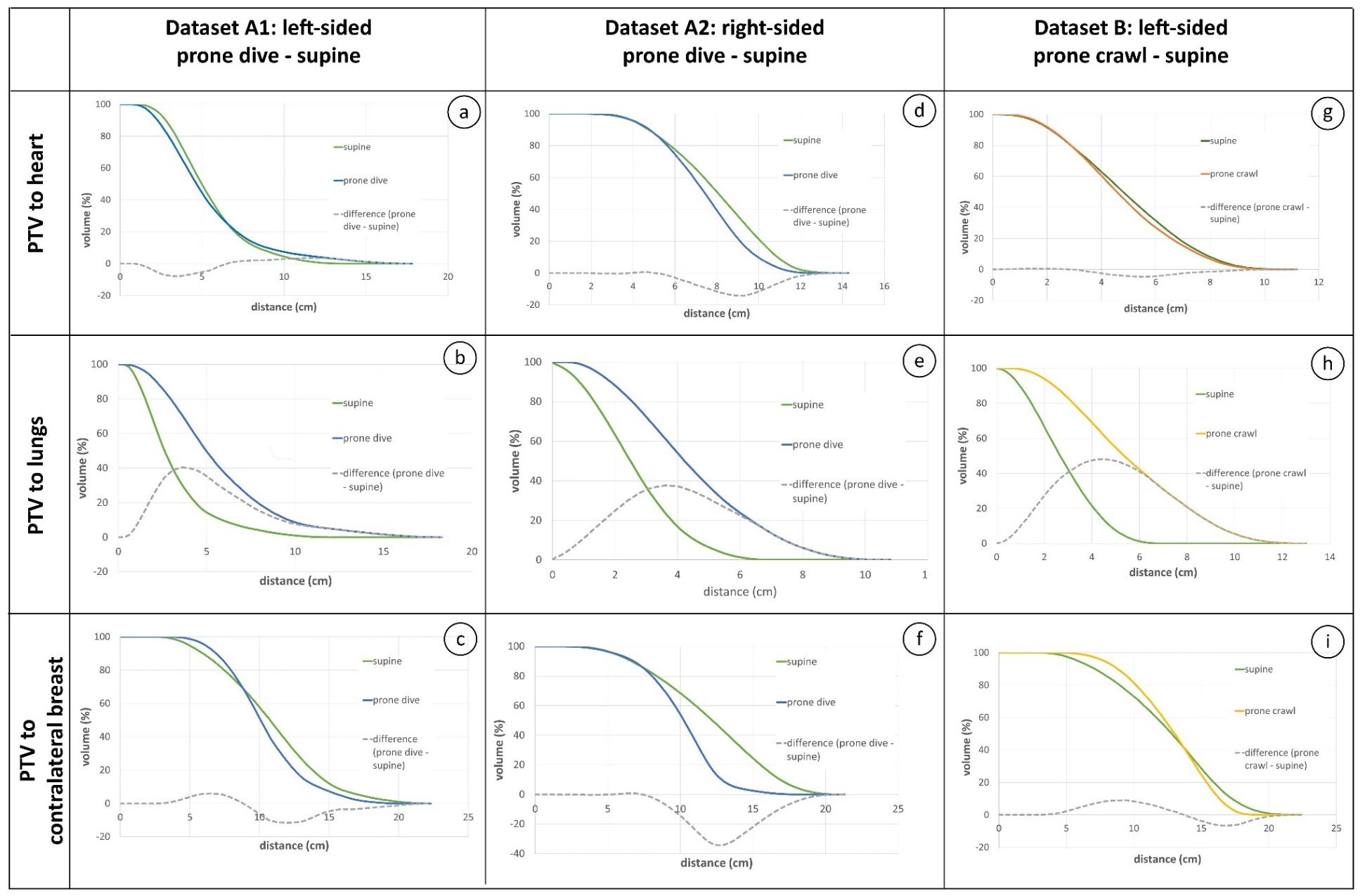

Supplement: Supplementary file 2 — FigureS02 [file ACM2-23-e13720-s002.doc]
