# Supplementary material for: Four irradiation and three positioning techniques for whole‐breast radiotherapy: Is sophisticated always better?
Source: J Appl Clin Med Phys. 2022 Sep 15;23(11):e13720. doi: 10.1002/acm2.13720 (PMC9680580; doi:10.1002/acm2.13720)
Supplement: Supplementary file 3 — TableS01 [file ACM2-23-e13720-s003.docx]

**Dose to organs-at-risk: extra structures**

**Dataset B-C-D: IL-arm (prone crawl)**

|  | **prone crawl** | | | | | | | | | |
| --- | --- | --- | --- | --- | --- | --- | --- | --- | --- | --- |
|  | **Dataset B (5L)** | | | **Dataset C (10R)** | | **Dataset D: SB (24L)** | | **Dataset D: DIBH (24L)** | | **Datasets B-D** |
|  | **TF-IMRT** | **MB-IMRT** | **IMAT** | **TF-IMRT** | **MB-IMRT** | **TF-IMRT** | **IMAT** | **TF-IMRT** | **IMAT** | **(all techniques)** |
| **IL arm: Dmax (Gy)** | 16.07 | 14.15 | 15.39 | 4.89 | 7.28 | 6.01 | 4.41 | 5.60 | 4.22 | 6.38 |
| **IL arm: V15Gy (cc)** | 0.00 | 0.06 | 0.14 | 0.01 | 0.00 | 0.00 | 0.00 | 0.00 | 0.00 | 0.01 |

**Dataset A1: left-sided (prone dive – supine)**

|  | **W-TF** | | | | | | **TF-IMRT** | | | | | | **MB-IMRT** | | | | | | | **IMAT** | | | | | | | |
| --- | --- | --- | --- | --- | --- | --- | --- | --- | --- | --- | --- | --- | --- | --- | --- | --- | --- | --- | --- | --- | --- | --- | --- | --- | --- | --- | --- |
|  | **prone dive** | | | **supine** | | | **prone dive** | | | **supine** | | | **prone dive** | | | **supine** | | | | **prone dive** | | | | **supine** | | | |
|  | dose (Gy) | range | SEM | dose (Gy) | range | SEM | dose (Gy) | range | SEM | dose (Gy) | range | SEM | dose (Gy) | range | SEM | dose (Gy) | range | SEM | dose (Gy) | | range | SEM | dose (Gy) | | range | SEM |  |
| **LNN_I (mean)** | **7.45** | (1.51-15.41) | 2.38 | **27.21** | (20.97-34.32) | 2.08 | **4.11** | (1.17-10.38) | 1.42 | **12.6** | (6.72-17.06) | 1.46 | **4.49** | (1.15-9.86) | 1.27 | **18.3** | (11.61-23.35) | 1.98 | **4.21** | | (1.33-8.81) | 1.14 | **14.94** | | (8.66-21.52) | 1.76 |  |
| **LNN_I (D02)** | **29.98** | (11.97-40.44) | 4.93 | **39.92** | (38.98-41.05) | 0.30 | **24.61** | (8.48-39.10) | 5.56 | **37.5** | (34.17-39.84) | 0.78 | **25.44** | (8.13-40.21) | 5.91 | **39.9** | (38.05-42.12) | 0.55 | **26.62** | | (9.30-42.67) | 5.99 | **38.08** | | (35.48-40.29) | 0.90 |  |
| **LNN_II (mean)** | **0.80** | (0.40-1.64) | 0.18 | **5.99** | (1.82-14.75) | 1.87 | **0.51** | (0.31-0.63) | 0.05 | **1.24** | (0.51-2.13) | 0.23 | **0.58** | (0.24-0.89) | 0.09 | **2.86** | (0.54-6.82) | 0.93 | **0.63** | | (0.28-0.97) | 0.10 | **1.46** | | (0.56-3.13) | 0.37 |  |
| **LNN_II (D02)** | **1.73** | (0.75-4.55) | 0.58 | **22.95** | (6.73-38.31) | 5.36 | **0.95** | (0.61-1.22) | 0.10 | **4.60** | (0.87-17.53) | 2.60 | **1.03** | (0.50-1.58) | 0.15 | **10.5** | (0.91-24.69) | 3.63 | **1.12** | | (0.55-1.67) | 0.17 | **5.99** | | (0.98-14.91) | 2.58 |  |
| **LNN_III (mean)** | **0.57** | (0.32-1.06) | 0.11 | **2.35** | (1.20-4.30) | 0.48 | **0.39** | (0.21-0.54) | 0.05 | **0.77** | (0.35-1.12) | 0.12 | **0.43** | (0.17-0.59) | 0.06 | **0.88** | (0.36-1.21) | 0.14 | **0.48** | | (0.19-0.67) | 0.07 | **0.74** | | (0.38-1.06) | 0.11 |  |
| **LNN_III (D02)** | **0.96** | (0.58-1.81) | 0.18 | **8.83** | (1.83-27.42) | 4.14 | **0.62** | (0.33-0.82) | 0.07 | **1.07** | (0.55-1.49) | 0.16 | **0.68** | (0.27-9.92) | 0.09 | **1.48** | (0.57-3.23) | 0.39 | **0.72** | | (0.30-0.95) | 0.09 | **1.09** | | (0.64-1.45) | 0.15 |  |
| **LNN_IV (mean)** | **0.35** | (0.17-0.69) | 0.08 | **0.97** | (0.58-1.35) | 0.10 | **0.26** | (0.16-.41) | 0.04 | **0.48** | (0.20-0.79) | 0.09 | **0.29** | (0.13-0.42) | 0.04 | **0.49** | (0.19-0.79) | 0.09 | **0.32** | | (0.13-0.53) | 0.05 | **0.43** | | (0.18-0.65) | 0.07 |  |
| **LNN_IV (D02)** | **0.52** | 0.26-0.98) | 0.11 | **1.35** | (0.84-1.92) | 0.15 | **0.40** | (0.27-0.57) | 0.05 | **0.69** | (0.29-1.13) | 0.12 | **0.45** | (0.21-0.73) | 0.07 | **0.71** | (0.29-1.12) | 0.12 | **0.48** | | (0.21-0.79) | 0.08 | **0.63** | | (0.29-0.96) | 0.10 |  |

**Dataset A2: right-sided (prone dive - supine)**

|  | **W-TF** | | | | | | **TF-IMRT** | | | | | | **MB-IMRT** | | | | | | **IMAT** | | | | | |
| --- | --- | --- | --- | --- | --- | --- | --- | --- | --- | --- | --- | --- | --- | --- | --- | --- | --- | --- | --- | --- | --- | --- | --- | --- |
|  | **prone dive** | | | **supine** | | | **prone dive** | | | **supine** | | | **prone dive** | | | **supine** | | | **prone dive** | | | **supine** | | |
|  | dose (Gy) | range | SEM | dose (Gy) | range | SEM | dose (Gy) | range | SEM | dose (Gy) | range | SEM | dose (Gy) | range | SEM | dose (Gy) | range | SEM | dose (Gy) | range | SEM | dose (Gy) | range | SEM |
| **LNN_I (mean)** | **7.48** | (2.78-15.57) | 1.96 | **35.24** | (25.88-40.19) | 2.17 | **6.23** | (1.94-13.50) | 1.61 | **27.59** | (19.67-39.22) | 2.99 | **4.41** | (1.25-7.69) | 1.07 | **28.90** | (22.96-37.14) | 2.03 | **4.13** | (1.46-7.70) | 0.85 | **22.08** | (9.55-32.75) | 3.53 |
| **LNN_I (D02)** | **34.81** | (26.76-38.75) | 1.75 | **42.05** | (41.00-43.56) | 0.39 | **30.03** | (12.75-39.04) | 3.88 | **41.99** | (38.62-43.73) | 0.78 | **24.91** | (6.06-38.21) | 5.27 | **41.32** | (38.14-43.19) | 0.76 | **25.99** | (7.95-35.80) | 4.38 | **39.31** | (34.60-42.39) | 1.09 |
| **LNN_II (mean)** | **0.86** | (0.45-1.52) | 0.18 | **23.68** | (9.21-39.31) | 5.60 | **0.89** | (0.56-1.67) | 0.18 | **12.33** | (2.84-36.82) | 5.14 | **0.80** | (0.52-1.34) | 0.14 | **11.38** | (5.45-23.61) | 2.63 | **0.75** | (0.53-1.12) | 0.09 | **7.16** | (2.21-14.49) | 2.00 |
| **LNN_II (D02)** | **1.89** | (0.88-3.33) | 0.45 | **38.21** | (35.25-41.63) | 0.88 | **2.34** | (1.08-5.83) | 0.79 | **31.61** | (19.95-40.88) | 3.23 | **2.23** | (0.98-6.28) | 0.86 | **30.56** | (17.90-38.09) | 3.53 | **1.44** | (0.95-2.05) | 0.19 | **21.08** | (7.33-38.12) | 4.65 |
| **LNN_III (mean)** | **0.63** | (0.34-0.98) | 0.11 | **15.47** | (2.50-39.13) | 5.84 | **0.65** | (0.39-1.06) | 0.11 | **7.40** | (1.06-35.93) | 5.71 | **0.60** | (0.38-0.83) | 0.08 | **5.56** | (1.13-22.65) | 3.44 | **0.60** | (0.42-0.82) | 0.08 | **3.91** | (0.94-16.68) | 2.56 |
| **LNN_III (D02)** | **1.03** | (0.65-1.50) | 0.15 | **27.25** | (7.50-40.52) | 5.60 | **0.98** | (0.68-1.42) | 0.12 | **9.89** | (1.47-40.55) | 6.23 | **0.92** | (0.63-1.23) | 0.10 | **9.87** | (1.49-36.53) | 5.45 | **0.93** | (0.62-1.17) | 0.09 | **7.35** | (1.22-31.75) | 4.90 |
| **LNN_IV (mean)** | **0.33** | (0.20-0.54) | 0.05 | **4.00** | (1.04-13.80) | 1.99 | **0.37** | (0.21-0.66) | 0.07 | **1.37** | (0.59-3.36 | 0.41 | **0.36** | (0.24-0.55) | 0.05 | **1.14** | (0.66-2.42) | 0.26 | **0.36** | (0.26-0.51) | 0.05 | **1.00** | (0.59-2.10) | 0.23 |
| **LNN_IV (D02)** | **0.50** | (0.31-0.74) | 0.08 | **10.52** | (1.39-36.27) | 5.71 | **0.54** | (0.37-0.86) | 0.08 | **3.58** | (0.86-14.89) | 2.26 | **0.52** | (0.36-0.71) | 0.06 | **2.08** | (0.93-6.09) | 0.81 | **0.52** | (0.41-0.68) | 0.05 | **1.69** | (0.82-4.82) | 0.63 |

**Dataset B: left-sided (prone crawl - supine)**

|  | **TF-IMRT** | | | | | | **MB-IMRT** | | | | | | **IMAT** | | | | | |
| --- | --- | --- | --- | --- | --- | --- | --- | --- | --- | --- | --- | --- | --- | --- | --- | --- | --- | --- |
|  | **prone crawl** | | | **supine** | | | **prone crawl** | | | **supine** | | | **prone crawl** | | | **supine** | | |
|  | dose (Gy) | range | SEM | dose (Gy) | range | SEM | dose (Gy) | range | SEM | dose (Gy) | range | SEM | dose (Gy) | range | SEM | dose (Gy) | range | SEM |
| **Esophagus (mean)** | **0.24** | (0.16-0.33) | 0.03 | **0.36** | (0.23-0.48) | 0.05 | **0.22** | (0.14-0.38) | 0.04 | **0.46** | (0.29-0.66) | 0.07 | **0.19** | (0.16-0.24) | 0.02 | **0.34** | (0.26-0.41) | 0.03 |
| **Esophagus (D02)** | **0.44** | (0.29-0.64) | 0.06 | **0.55** | (0.36-0.79) | 0.07 | **0.44** | (0.26-0.86) | 0.11 | **0.70** | (0.46-1.06) | 0.11 | **0.35** | (0.29-0.41) | 0.02 | **0.54** | (0.40-0.67 | 0.05 |
| **LNN_I (mean)** | **3.29** | (1.99-5.90) | 0.76 | **28.81** | (25.99-33.09) | 1.22 | **6.96** | (3.81-13.78) | 1.97 | **35.53** | (33.93-37.58) | 0.78 | **6.30** | (1.69-12.81) | 2.26 | **30.44** | (28.01-34.51) | 1.10 |
| **LNN_I (D02)** | **29.72** | (21.66-39.37) | 3.76 | **40.45** | (39.81-40.79) | 0.17 | **27.06** | (8.92-35.73) | 5.07 | **42.48** | (40.20-43.24) | 0.58 | **25.49** | (10.05-37.30) | 4.76 | **41.65** | (40.22-42.68) | 0.45 |
| **LNN_II (mean)** | **0.74** | (0.50-0.96) | 0.08 | **8.39** | (4.31-16.72) | 2.18 | **0.90** | (0.65-1.15) | 0.08 | **14.86** | (8.85-21.72) | 2.66 | **0.87** | (0.57-1.13) | 0.12 | **7.62** | (4.51-14.16) | 1.83 |
| **LNN_II (D02)** | **1.51** | (1.06-2.04) | 0.21 | **25.68** | (18.24-35.86) | 2.97 | **3.36** | (1.68-6.17) | 0.87 | **35.49** | (32.68-40.31) | 1.42 | **2.16** | (1.09-3.51) | 0.45 | **23.90** | (18.53-35.34) | 3.09 |
| **LNN_III (mean)** | **0.44** | (0.35-0.65) | 0.06 | **2.72** | (1.31-6.17) | 0.90 | **0.43** | (0.26-0.60) | 0.06 | **4.37** | (1.53-12.26) | 1.99 | **0.45** | (0.29-0.67) | 0.07 | **1.75** | (1.24-2.21) | 0.20 |
| **LNN_III (D02)** | **0.73** | (0.62-0.95) | 0.06 | **9.98** | (1.86-26.41) | 4.43 | **0.70** | (0.53-0.85) | 0.06 | **12.67** | (2.28-32.77) | 5.36 | **0.75** | (0.60-0.98) | 0.07 | **4.72** | (2.14-8.70) | 1.27 |
| **LNN_IV (mean)** | **0.24** | (0.14-0.37) | 0.04 | **1.94** | (0.56-6.35) | 1.10 | **0.23** | (0.09-0.35) | 0.04 | **2.17** | (0.66-6.77) | 1.16 | **0.22** | (0.13-0.32) | 0.03 | **1.66** | (0.59-5.28) | 0.91 |
| **LNN_IV (D02)** | **0.35** | (0.21-0.56) | 0.06 | **8.97** | (0.87-40.35) | 7.85 | **0.33** | (0.17-0.52) | 0.06 | **9.75** | (0.97-43.15) | 8.35 | **0.33** | (0.19-0.52) | 0.06 | **8.23** | (0.89-37.02) | 7.20 |

**Dataset C: right-sided (prone crawl – prone dive)**

|  | **TF-IMRT** | | | **MB-IMRT** | **prone crawl** |
| --- | --- | --- | --- | --- | --- |
|  | **prone crawl** | **prone dive** |  | **prone crawl** | **TF-IMRT/MB-IMRT** |
|  | dose (Gy) | dose (Gy) | p-value | dose (Gy) | p-value |
| Heart (mean) | **0.50** | **0.64** | <0.01 | **0.45** | 0.02 |
| apex heart (mean) | **0.39** | **0.49** | 0.02 | **0.37** | 0.26 |
| LAD (mean) | **0.43** | **0.59** | <0.01 | **0.39** | 0.03 |
| CL lung (left) (mean) | **0.08** | **0.12** | <0.01 | **0.08** | 0.03 |
| IL Lung (right) (mean) | **0.63** | **1.36** | 0.01 | **0.58** | 0.32 |
| Lungs (mean) | **0.37** | **0.72** | 0.01 | **0.34** | 0.32 |
| CL breast (mean) | **0.36** | **0.60** | <0.01 | **0.36** | 0.92 |
| CL breast (D02) | **1.33** | **1.77** | 0.03 | **1.34** | 0.86 |

Extra table with paired t-test for n=9 instead of n=10 (Table 8). For one patient in the prone crawl position of dataset C, an exceptionally high extent of the lateral part of the CTV_WBI is present in posterior direction, while this is not the case in the prone dive position of the same patient. This resulted in dose endpoints of OARs for this dataset being non-normally distributed, and in a failure to have significance on dose endpoints for heart, apex and CL breast between prone dive and prone supine positions. In the above table, we have excluded this patient and performed a paired t-test on a normally distributed dataset of n=9. The difference with Table 8 is most apparent with the heart, apex and CL breast in TF-IMRT. With a paired t-test on 9 subjects in TF-IMRT, the prone crawl position significantly reduces dose to cardiac structures and CL breast for this selection of patients. For the cardiac structures, the differences are relatively small, but for the CL breast there is almost 50% reduction.

**Dataset C: right-sided (prone crawl – prone dive)**

|  | **TF-IMRT** | | | **MB-IMRT** | **prone crawl** |
| --- | --- | --- | --- | --- | --- |
|  | **prone crawl** | **prone dive** | p-value | **prone crawl** | **TF-IMRT/MB-IMRT** |
|  | dose (Gy) | dose (Gy) |  | dose (Gy) | p-value |
| **Esophagus (mean)** | **0.14** | **0.26** | 0.01 | **0.13** | 0.06 |
| **Esophagus (D02)** | **0.26** | **0.37** | 0.01 | **0.23** | 0.01 |
| **LNN_I (mean)** | **3.66** | **10.55** | 0.01 | **5.17** | 0.02 |
| **LNN_I (D02)** | **24.40** | **33.93** | 0.01 | **24.69** | 0.96 |
| **LNN_II (mean)** | **0.68** | **1.29** | 0.01 | **0.78** | 0.51 |
| **LNN_II (D02)** | **1.88** | **4.00** | 0.51 | **2.47** | 0.33 |
| **LNN_III (mean)** | **0.34** | **0.87** | 0.01 | **0.31** | 0.05 |
| **LNN_III (D02)** | **0.59** | **1.29** | 0.01 | **0.52** | 0.02 |
| **LNN_IV (mean)** | **0.20** | **0.53** | 0.01 | **0.18** | 0.01 |
| **LNN_IV (D02)** | **0.30** | **0.70** | 0.01 | **0.27** | 0.01 |

**Dataset D: left sided prone crawl (DIBH – SB)**

|  | **prone crawl** | | | | | | | | |
| --- | --- | --- | --- | --- | --- | --- | --- | --- | --- |
|  | **TF-IMRT** | | | **IMAT** | | | **DIBH** | **SB** | |
|  | **DIBH** | **SB** | p-value | **DIBH** | **SB** | p-value | **TF-IMRT/IMAT** | **TF-IMRT/IMAT** |  |
|  | dose (Gy) | dose (Gy) |  | dose (Gy) | dose (Gy) |  | p-value | p-value |  |
| **Thyroid (mean)** | **0.08** | **0.09** | <0.01 | **0.09** | **0.10** | 0.02 | <0.01 | 0.41 |  |
| **Brachial Plexus (D02)** | **0.35** | **0.44** | <0.01 | **0.30** | **0.50** | 0.12 | <0.01 | 0.64 |  |
| **Spinal cord (D02)** | **0.12** | **0.12** | 0.06 | **0.11** | **0.12** | 0.33 | 0.29 | 0.20 |  |
| **LNN_I (mean)** | **3.78** | **4.55** | 0.03 | **3.51** | **3.63** | 0.70 | 0.42 | <0.01 |  |
| **LNN_I (D02)** | **26.96** | **28.25** | 0.32 | **25.33** | **26.09** | 0.47 | 0.12 | 0.01 |  |
| **LNN_II (mean)** | **0.49** | **0.57** | <0.01 | **0.46** | **0.47** | 0.77 | 0.26 | <0.01 |  |
| **LNN_II (D02)** | **1.28** | **1.56** | 0.04 | **1.43** | **1.28** | 0.49 | 0.47 | 0.08 |  |
| **LNN_III (mean)** | **0.28** | **0.34** | <0.01 | **0.26** | **0.29** | 0.01 | 0.18 | <0.01 |  |
| **LNN_III (D02)** | **0.48** | **0.56** | <0.01 | **0.46** | **0.49** | 0.03 | 0.17 | <0.01 |  |
| **LNN_IV (mean)** | **0.15** | **0.19** | <0.01 | **0.15** | **0.17** | <0.01 | 0.49 | 0.03 |  |
| **LNN_IV (D02)** | **0.24** | **0.29** | <0.01 | **0.23** | **0.26** | <0.01 | 0.26 | <0.01 |  |
